# Natural Organic Matter Stabilizes Pristine Nanoplastics but Destabilizes Photochemical Weathered Nanoplastics in Monovalent Electrolyte Solutions

**DOI:** 10.1021/acs.est.4c11540

**Published:** 2025-01-15

**Authors:** Yanghui Xu, Xintu Wang, Jan Peter van der Hoek, Gang Liu, Kim Maren Lompe

**Affiliations:** † Key Laboratory of Drinking Water Science and Technology, Research Centre for Eco-Environmental Sciences, Chinese Academy of Sciences, Beijing 100085, P. R. China; ‡ Section of Sanitary Engineering, Department of Water Management, Faculty of Civil Engineering and Geosciences, Delft University of Technology, Stevinweg 1, 2628 CN Delft, The Netherlands; § Waternet, Department Research & Innovation, P.O. Box 94370, 1090 GJ Amsterdam, The Netherlands; ∥ University of Chinese Academy of Sciences, Beijing 100049, China

**Keywords:** nanoplastics, aggregation tendency, natural organic matter, photochemical weathering, steric repulsion, polymer bridging

## Abstract

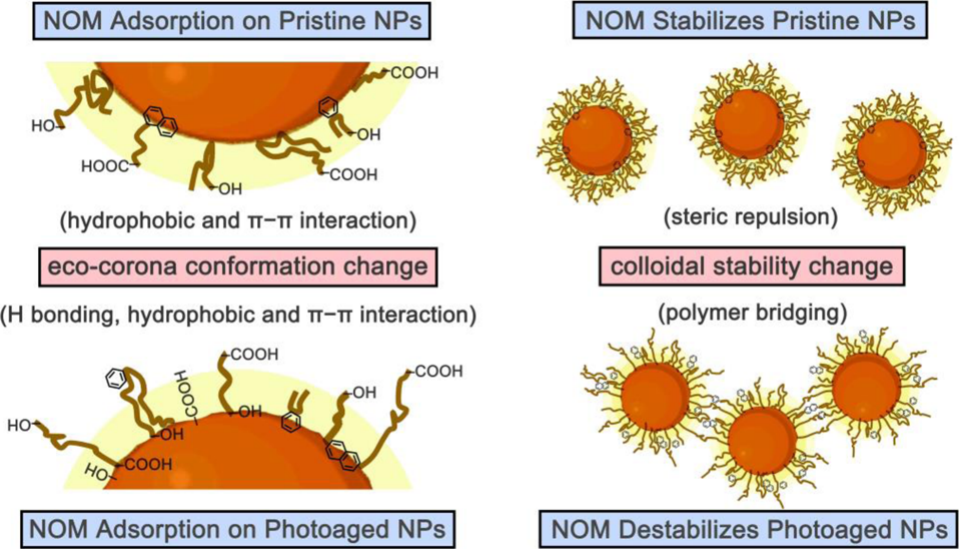

Photochemical weathering and eco-corona formation through natural organic matter (NOM) adsorption play vital roles in the aggregation tendencies of nanoplastics (NPs) in aquatic environments. However, it remains unclear how photochemical weathering alters the adsorption patterns of NOM and the conformation of the eco-corona, subsequently affecting the aggregation tendencies of NPs. This study examined the effect of Suwannee River NOM adsorption on the aggregation kinetics of pristine and photoaged polystyrene (PS) NPs in monovalent electrolyte solutions. The results showed that photochemical weathering influenced the conformation of the eco-corona, which, in turn, determined NP stability in the presence of NOM. Hydrophobic components of NOM predominantly bound to pristine NPs through hydrophobic and π–π interactions, and extended hydrophilic segments in water hindered NP aggregation via steric repulsion. Conversely, hydrogen bonding facilitated the binding of these hydrophilic segments to multiple photoaged NPs, thereby destabilizing them through polymer bridging. Additionally, the stabilization and destabilization capacities of NOM increased with its concentration and molecular weight. These findings shed light on the destabilizing role of NOM in weathered NPs, offering new perspectives on environmental colloidal chemistry and the fate of NPs in complex aquatic environments.

## Abstract 2

Photochemical weathering of nanoplastics (NPs) generated heterogeneous surfaces that facilitated NOM adsorption through multiple forces, enabling NOM bridging and leading to the destabilization of the weathered NPs.

## Introduction

The extensive production and widespread use of plastics contribute substantially to the accumulation of plastic debris in terrestrial and aquatic ecosystems, comprising a substantial portion of marine litter, ranging from 60% to 80%.^1−83^ Over time, these plastic materials undergo a series of processes such as chemical degradation, biodegradation, photodegradation, thermal degradation, and mechanical abrasion, breaking them down into minute particles at the nanoscale (<1 μm) termed as nanoplastics (NPs).^1,5−8^ Once entering the aquatic environment, NPs may be exposed to a series of physicochemical processes, such as sunlight-induced photooxidation,^9^ aggregation,^10,11^ deposition,^12,13^ and adsorption,^14,15^ which are closely linked to their fate, bioavailability, and biotoxicity.^1,16^

The colloidal stability and aggregation tendencies of NPs have emerged as a recent focal point of research interest, often drawing insights from the chemistry of other colloids such as natural colloids and engineered TiO_2_ nanoparticles.^17,18^ The aggregation or flocculation of colloids is typically controlled by soluble polymers, such as engineered synthetic polymers like polyethylene glycol and polyacrylamide.^19,20^ Similarly, in environmental systems, natural polymer such as natural organic matter (NOM) plays a vital role in the aggregation of colloids and nanoparticles.^21^ The role of soluble polymers in the aggregation of nanoparticles is often studied in monovalent electrolyte solutions.^22−24^ These soluble polymers can induce either attractive or repulsive interactions depending on their adsorption capacity on the nanoparticle surface.^19^ If the polymer fails to absorb onto the nanoparticle surface, the exclusion of polymers from the space between the nanoparticles can create osmotic forces known as depletion attractions, which push larger nanoparticles together and promote their aggregation.^25^ On the other hand, if the polymer fully coats the nanoparticle surface, it can stabilize the nanoparticles through steric repulsion.^22^ However, if the added polymer adheres to the colloid surface, various segments of the same polymer may attach to different nanoparticles, leading to particle aggregation via polymer bridging.^21^ In environmental systems, the stabilization of nanoparticles by NOM through steric repulsion typically occurs,^23,24^ while destabilization via depletion attraction or polymer bridging is less observed.^26−29^ One study demonstrated that citrate-stabilized Au nanoparticles were destabilized by fulvic acid (FA) via bridging flocculation driven by hydrophobic interactions between adsorbed FA molecules.^28^ In some cases, destabilization of nanoparticles by NOM might occur via electrostatic patch-charge attraction if the nanoparticles (e.g., ferrihydrite and TiO_2_) and NOM have opposite charges^30,31^ and via cation bridging in the presence of multivalent cations, such as calcium ions.^32,33^

NOM, ubiquitous natural molecules in aquatic environments, consists of heterogeneous organic compounds, including humic acids (HA), FA, proteins, and polysaccharides.^34,35^ NPs and NOM in environments can interact through diverse attractions such as van der Waals interactions, electrostatic interactions, hydrophobic interactions, hydrogen bonding, and π–π interactions.^36,37^ The adsorption of NOM on NPs often results in the formation of an organic coating on the NP surface, referred to as an eco-corona.^38^ This eco-corona can alter the physicochemical properties of NPs, affecting their aggregation behaviors in aquatic environments.^34^ The eco-corona often enhances the stability of NPs in monovalent electrolyte solutions, primarily due to steric repulsion.^11,37,39^ Photochemical weathering is another key process that modifies the surface of NPs, typically rendering them more hydrophilic and negatively charged.^13,40^ By altering the interactions between NOM and NPs, photochemical weathering can influence the formation and characteristics of the eco-corona.^36^ Using a quartz crystal microbalance with dissipation, Schefer et al. indicated that photochemical weathering decreased the adsorbed mass of HA, FA, and Suwannee River NOM on plastic surfaces in synthetic freshwater.^36^ Additionally, studies demonstrated that photooxidation resulted in a decrease in the hydrophobic and π–π interactions between polystyrene (PS) NPs and HA, consequently weakening the stabilization capacity of HA on PS NPs in monovalent solutions.^37,41^ These findings provide insights into the stabilizing effect of the eco-corona on both pristine NPs and those subjected to photochemical weathering.

Nevertheless, NOM comprises chemically diverse polymers with varying molecular weights (MWs) and chemical structures.^34,42,43^ The high-MW fraction of NOM typically consists of abundant hydrophobic and aromatic components, whereas the low-MW fraction contains a higher proportion of hydrophilic O-containing groups.^42,44^ By influencing the adsorption pattern of NOM on NPs, photochemical weathering has the potential to change not only the mass/thickness but also the fractionation and spatial arrangement of the eco-corona on NPs^45^ and potentially leads to distinct stability outcomes. For instance, the photochemical weathered NPs might not adsorb specific fractions of NOM, leading to depletion attraction and potential destabilization of the NPs. Alternatively, hydrogen bonding might dominate the adsorption of NOM on weathered NPs, allowing various hydrophilic segments of the NOM to attach to different NPs, thereby destabilizing NPs via polymer bridging. Further investigation is necessary to determine whether these processes could occur and have distinct effects on the NP stability.

The main purpose of this study is to investigate the interaction between NOM and pristine/photoaged NPs and their effects on NP aggregation. To address this knowledge gap, the aggregation tendencies of pristine and photochemically weathered PS NPs were studied both in the absence and presence of Suwanee River NOM, as well as NOM fractions fractionated by MW. PS NPs were aged using the artificial accelerated photooxidation method to harvest photoaged NPs. Bulk NOM (<0.05 μm) and NOM fractions (<3 kDa, 3–10 kDa, 10–30 kDa, and 30 kDa–0.05 μm) were prepared for adsorption tests in stirred batch reactors using pristine and photoaged NPs. Time-resolved dynamic light scattering (DLS) was used to observe the effects of bulk NOM and NOM fractions on the aggregation kinetics of pristine and photoaged NPs in sodium chloride solutions.

## Materials and Methods

### Materials

A PS stock solution with a concentration of 50% (w/v) and an NP nominal size of 100 nm was purchased from Zhichuan Intelligent Technology (Suzhou) Co., Ltd. PS NPs were chosen for their occurrence in natural waters,^46^ and widespread use in aggregation studies,^7,9,41^ facilitating comparison with existing research. According to the manufacturer, the PS NPs were unfunctionalized but contained the surfactant sodium dodecyl sulfate (SDS). To reduce the interference with this surfactant, the prepared PS suspension (5 g/L) was washed four times using ultrapure water through ultracentrifugation (22,000*g*, 30 min) and sonication (40 kHz, 5 min) until the total organic carbon (TOC) in the supernatant was negligible (0.38 ± 0.11 mg C/L). The hydrodynamic sizes of unwashed and washed NPs were measured as 161.5 ± 1.3 and 159.8 ± 0.7 nm, with polydispersity indices of 0.063 and 0.057, respectively, indicating that the cleaning process did not induce aggregation of PS NPs. Suwannee River NOM was purchased from the International Humic Substances Society. The poly(vinylidene fluoride) (PVDF) ultrafiltration membranes were purchased from RisingSun Membrane Technology (Beijing) Co., Ltd.

### NOM Fractions Preparation

To prepare the NOM stock solution, 200 mg of NOM was dissolved in 200 mL of ultrapure water under sonication. The pH was then adjusted to 10 using 0.1 M NaOH to enhance NOM dissolution and prevent aggregation.^36^ The solution underwent stirring at 200 rpm for 24 h to facilitate solubilization. A stainless steel Amicon stirred cell (100 mL) ultrafiltration system equipped with a 0.05 μm membrane disc (PVDF) was employed to remove any undissolved NOM and collect bulk NOM. NOM with a size below 0.05 μm, which accounts for the majority of NOM,^47,48^ was selected for easy separation from the larger NPs (100 nm) after the adsorption experiment using 0.1 μm filters. Following this procedure, NOM fractions were separated via successive filtration through 30, 10, and 3 kDa membrane discs (PVDF) to collect NOM fractions with MWs ranging from 30 kDa to 0.05 μm, 10 to 30 kDa, 3 to 10 kDa, and <3 kDa. After the initial ultrafiltration, the membrane was rinsed with 5 mL of ultrapure water and then subjected to filtration again. This procedure was repeated twice to achieve improved separation performance and minimize loss. The concentrated retentate was collected and then diluted with ultrapure water. The harvested NOM fractions were filtered using 0.1 μm PES filters for further use, aimed at removing any particulates resulting from the ultrafiltration procedure. The TOC concentrations of the final bulk NOM and NOM fractions measured with TOC analysis (Shimadzu, ASI-V) were as follows: 343 mg/L for bulk NOM (<0.05 μm), 244 mg/L for NOM (30 kDa–0.05 μm), 101 mg/L for NOM (10–30 kDa), 243 mg/L for NOM (3–10 kDa), and 131 mg/L for NOM (<3 kDa). The entire procedure resulted in a 12.2% loss, with the proportions being 39.2% (<3 kDa), 17.1% (3–10 kDa), 6.8% (10–30 kDa), and 19.2% (30 kDa–0.05 μm). The resulting pH of the stock solutions was 7.1 for bulk NOM, 7.6 for <3 kDa, 7.1 for 3–10 kDa, 7.2 for 10–30 kDa, and 7.2 for 30 kDa–0.05 μm fractions. These NOM solutions were diluted to achieve a consistent DOC concentration of 1–10 mg/L for the adsorption experiments and the aggregation experiments.

### Accelerated Photooxidation Experiments

A mercury lamp (500 W) emitting UV light with an intensity of approximately 35 mW/cm^2^ was employed to age the PS NPs. The spectrum of the mercury lamp is shown in Figure S1. The accelerating factor of the lamp was assessed by comparing the aging extent of PS NPs under real sunlight exposure in The Netherlands (see details in Text S1 and Figures S1 and S2). Pristine PS NPs, with a concentration of 300 mg/L (200 mL), were introduced into a 300 mL transparent quartz reactor placed on a magnetic stirrer. The lamp, positioned within a circulation-cooled quartz tube, was placed inside the quartz reactor. The PS NPs were aged for 2, 4, and 8 days, corresponding to approximately 40, 80, and 160 days of sunlight exposure in The Netherlands. The particle number concentrations of both pristine and photoaged NPs were considered the same as photoaging did not cause obvious fragmentation of NPs (see Results and Discussion). To eliminate the interference of the leached dissolved organic carbon from plastic photodegradation in subsequent experiments, the aged NPs were washed four times using ultrapure water through ultracentrifugation (Eppendorf, Centrifuge 5910 Ri) at 15,000 rpm (22,000*g*) for 30 min and sonication (DK-3000H) at 40 kHz for 5 min. The washed pristine and aged NPs for 2, 4, and 8 days were labeled as PS_0_, PS_2_, PS_4_, and PS_8_, respectively (Figure S3). Due to changes in NP concentration during the washing process, the concentration of washed aged NPs was determined using UV absorbance at 289 nm and adjusted to match the UV_289_ of the unwashed aged NPs. For subsequent adsorption and aggregation experiments, approximately 4.68 × 10^12^ particles/L of pristine (10 mg/L) and photoaged NPs were used by diluting the stock solutions to match their corresponding UV_289_ (0.952, 0.927, 0.916, and 0.902 for PS_0_, PS_2_, PS_4_, and PS_8_, respectively). The dilution resulted in final pH values of 5.7, 5.9, 5.8, and 6.0 for PS_0_, PS_2_, PS_4_, and PS_8_, respectively. The corresponding TOC concentrations for PS_0_, PS_2_, PS_4_, and PS_8_ were measured as 29.2 mg C/L, 26.8 mg C/L, 24.7 mg C/L, and 23.6 mg C/L, respectively (Table S1). High concentrations of the NPs were used for a better observation of experimental phenomena.

### Adsorption Experiments

Batch adsorption experiments were carried out to examine the adsorption of bulk NOM and NOM fractions onto pristine and photoaged NPs. Pristine or photoaged NPs (10 mg/L) were mixed with bulk NOM or NOM fractions (2 mg/L) in 10 and 100 mM NaCl solutions within 50 mL centrifuge tubes. At these NaCl concentrations, no aggregation occurred, making them suitable for testing the NOM adsorption. The pH was adjusted to 6.0 ± 0.1 using 0.1 M HCl and NaOH to reflect environmentally relevant conditions.^11,52^ The centrifuge tubes were agitated at 100 rpm in a shaker at room temperature for 24 h (preliminary tests indicated that equilibrium was reached after this duration).^53^ Following this, the solution was filtered through 0.1 μm PES filters (prewashed with 10 mL of ultrapure water) to separate NPs and NOM. For each solution, the first 3 mL of filtrate was discarded, and the remaining filtrate was used to test NOM removal. Preliminary experiments indicated that NOM adsorption on filters was negligible, and NOM adsorption by NPs had minimal impact on the specific absorbance of its UV spectra (Figure S4). The concentrations of NOM before and after adsorption were determined using UV absorbance at 280 nm, an index that reflects the aromaticity of NOM and is commonly used for its quantification.^30,43,54^ Control samples of the washed NPs were compared to a blank sample, confirming no contributions from the NPs to the UV absorbance in the filtrate (Figure S5). Duplicates were performed to assess the reduction in the NOM concentration (1 – *A*/*A*_0_) after adsorption onto pristine and photoaged NPs (*A*_0_ and *A* mean UV absorbance at 280 nm before and after adsorption).

### Aggregation Kinetics Measurements

The time-resolved DLS technique was used to examine the aggregation kinetics of NPs in NaCl solutions utilizing a Malvern Zetasizer instrument (Nano ZS, Malvern, UK). The NOM and NP samples were premixed in ultrapure water, and the pH was adjusted to 6.0 ± 0.1 using 0.1 M HCl and NaOH. The concentration of NPs was 10 mg/L, a common concentration for aggregation kinetic studies.^9,11,52,55^ Bulk NOM (<0.05 μm) was adjusted to concentrations ranging from 1 to 10 mg C/L to examine the effect of NOM concentration on NP aggregation, within the typical range found in natural waters.^11,39,56^ To further investigate the impact of the NOM MW, a representative concentration of 2 mg/L was selected for different NOM fractions. The concentration of monovalent electrolyte solution (NaCl) ranged from 100 to 1000 mM.^57^ A relatively wide range of NaCl concentrations were tested to estimate the critical coagulation concentration (CCC) values and gain a better understanding of the aggregation tendencies of NPs. Duplicates or triplicates were conducted for each sample. Details on the detection and calculation for the aggregation kinetics can be found in the Supporting Information (Text S2). The Zetasizer instrument provided the intensity-weighted hydrodynamic size of the NPs. To gain more insights into the aggregation kinetics, the intensity-, volume-, and number-weighted hydrodynamic sizes were analyzed using the Litesizer DLS 700 instrument (Anton Paar, Austria).

### Characterization

UV–vis spectroscopy (G10S UV–vis, Thermo Fisher Scientific) and three-dimensional excitation–emission matrix (3D-EEM) fluorescence spectroscopy (Horiba Scientific) were employed to characterize the chemical properties of bulk NOM and NOM fractions. The hydrodynamic size and zeta potential of NPs in the absence and presence of NOM were measured using a Zetasizer Nano ZS90 (Malvern Instruments, UK). The size and morphology of pristine/photoaged NPs in ultrapure water were examined using scanning electron microscopy (SEM, Quattro, FEI) and transmission electron microscopy (TEM, Tecnai G20, FEI Corp, USA). Attenuated total reflectance Fourier transform infrared spectroscopy (ATR-FTIR, Nicolet iN10, Thermo Fisher Scientific) was used to detect the surface functional groups of NPs and NOM, as well as their interactions.^7^ Detailed characterization methods are provided in Text S3.

### DLVO and Steric (Polymer-Mediated) Interaction Energy

The classical Derjaguin–Landau–Verwey–Overbeek (DLVO) theory was used to describe the interaction forces between NPs and includes van der Waals and electrostatic double layer interactions.^23,58^ In the presence of NOM, the extended DLVO (XDLVO) theory considering a steric (polymer-mediated) model was used to investigate interaction energies between NPs. This model takes into account repulsive steric interaction that arises when the adsorbed NOM forms a brush-like layer around the NPs, as well as attractive steric interaction (i.e., polymer bridging) that can occur when the polymer is able to adsorb onto multiple NPs simultaneously.^20,59^ Detailed calculations of DLVO and polymer-mediated interaction energies are provided in Text S4 and Tables S2–S5.

## Results and Discussion

### Photochemical Weathering Modifies NPs

**Figure 1:**
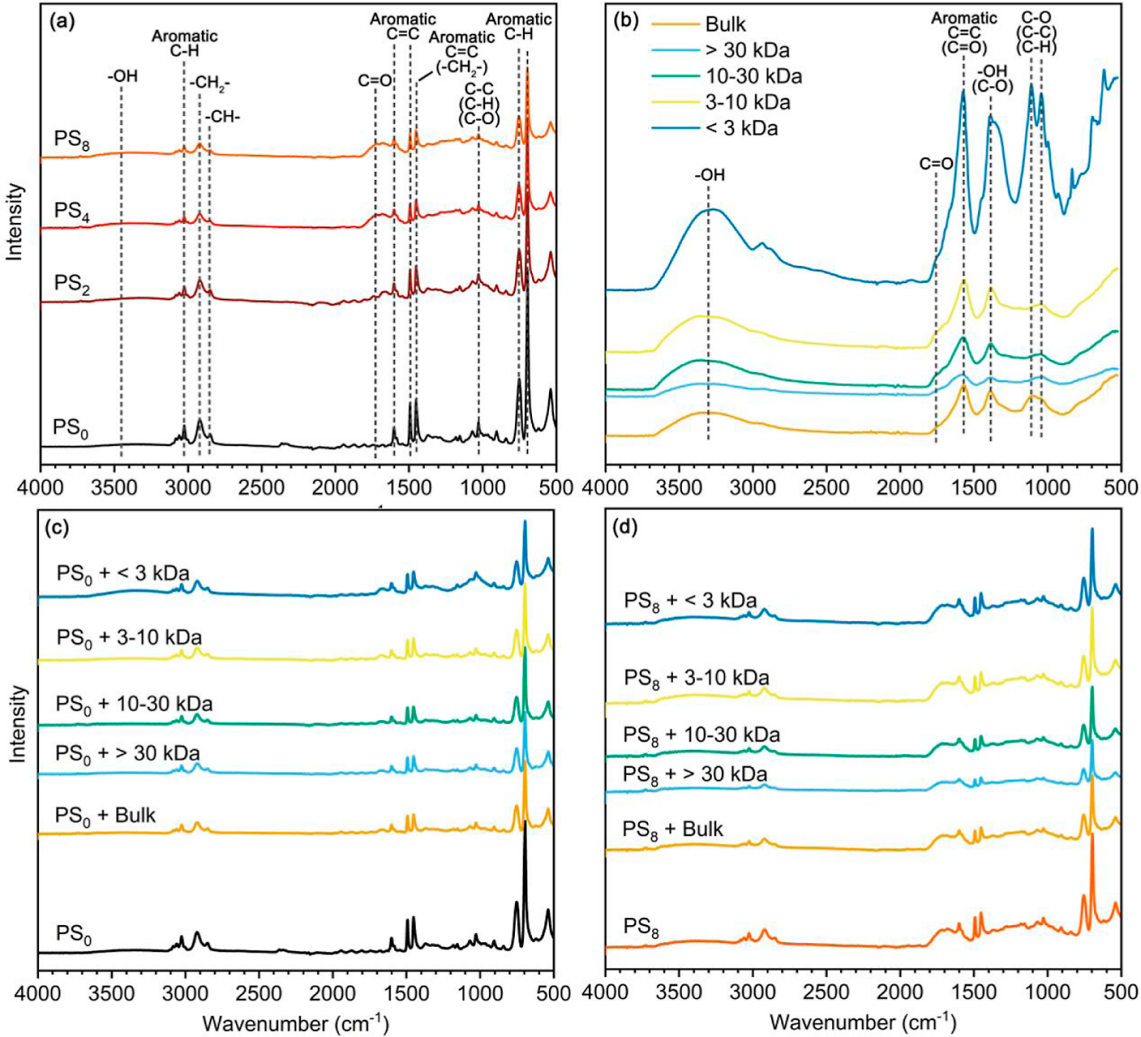
FTIR spectra of pristine and photoaged NPs (a), bulk NOM and NOM fractions (b), PS_0_ before and after adsorption with bulk NOM and NOM fractions in 100 mM NaCl (c), and PS_8_ before and after adsorption with bulk NOM and NOM fractions at 100 mM NaCl. Subscripts 0, 2, 4, and 8 mean aging times of 0, 2, 4, and 8 d.

**Figure 2:**
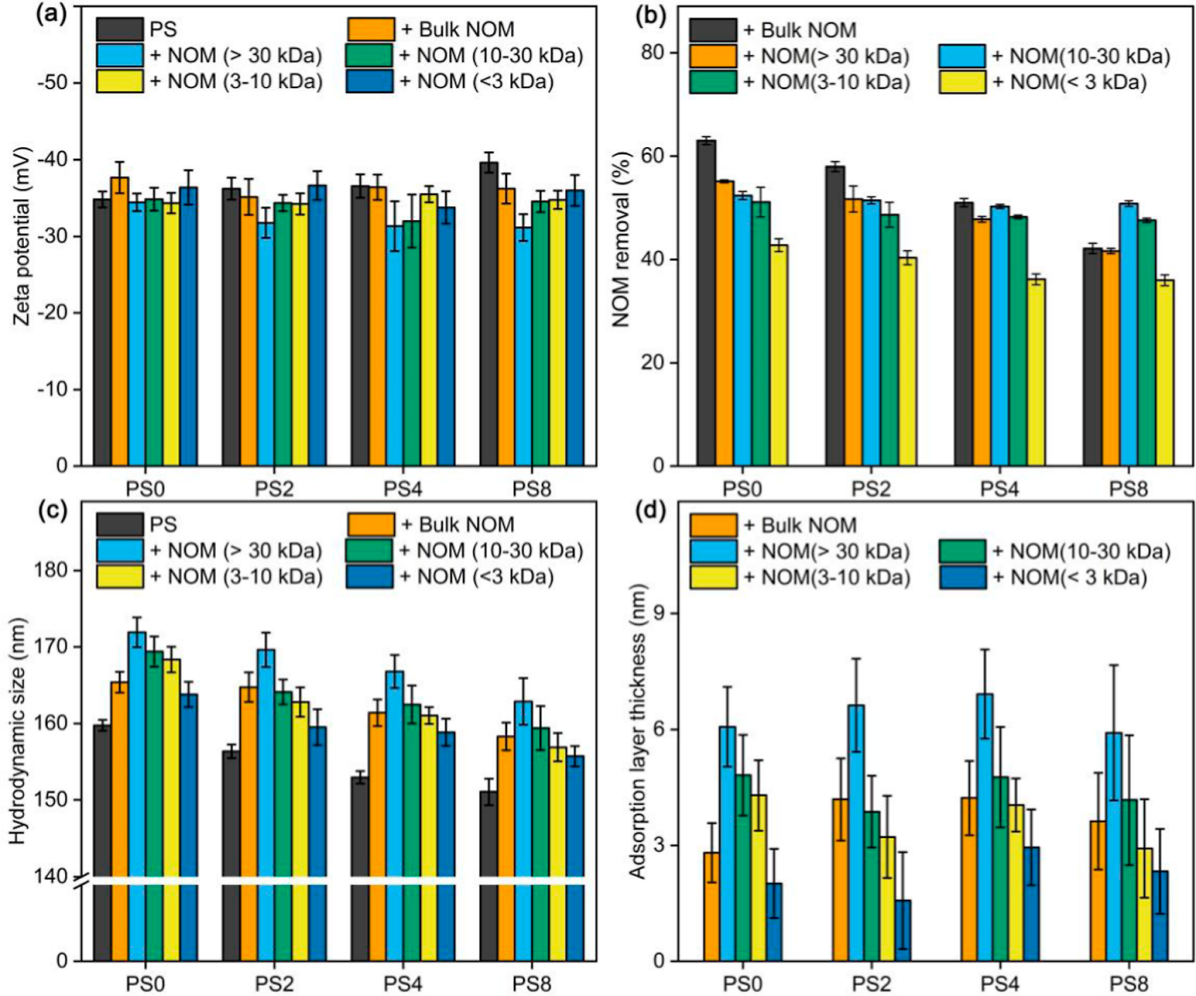
(a) Zeta potential of pristine and photoaged NPs with and without bulk NOM and NOM fractions (2 mg/L) in 100 mM NaCl after 24 h (*n* = 10). (b) Removal (UV_280_ reduction) of bulk NOM and NOM fractions (2 mg/L) after adsorption (24 h) on pristine and photoaged NPs in 100 mM NaCl (*n* = 2). (c) Hydrodynamic size of pristine and photoaged NPs with and without bulk NOM and NOM fractions (2 mg/L) in 100 mM NaCl after 24 h (*n* = 10) (d) Adsorption layer thickness of bulk NOM and NOM fractions (2 mg/L) on pristine and photoaged NPs in 100 mM NaCl after 24 h. Error bars represent the mean ±1.96 SE (95% confidence interval). Subscripts 0, 2, 4, and 8 mean aging times of 0, 2, 4, and 8 d.

The FTIR spectra and the corresponding functional groups with their respective absorption wavenumbers are presented in Figure 1a and Table S6. As the photoaging time was prolonged, a decrease in the intensity of peaks at 696, 753, 1028, 1452, 1494, 1601, 2850, 2920, and 3026 cm^–1^ was observed, indicating the degradation of aliphatic segments and benzene rings of pristine PS NPs under UV exposure. Notably, the new peaks at around 1717 and 3453 cm^–1^ appeared after the photochemical weathering of PS NPs, attributed to the stretching vibrations of carboxyl (C=O) and hydroxyl (−OH) groups.^60^ This is consistent with previous studies indicating that photochemical weathering can degrade initial hydrophobic aromatic and aliphatic components while generating hydrophilic O-containing functional groups.^16,37^ PS NPs had a specific UV absorbance at around 289 nm, which is attributed to the π–π* transition of the benzenoid ring.^49^ Photochemical weathering led to a reduction in UV absorbance at 289 nm (Table S1),^50^ likely due to the destruction of the benzene ring structure.^51^ The initial zeta potential of the pristine NPs (washed to remove SDS as described in the methods) was already low (−50.3 ± 1.4 mV in 10 mM NaCl), most likely due to sulfate groups generated during their synthesis.^9,51^ The zeta potential decreased further as a result of aging to −63.8 ± 0.8 mV, −65.7 ± 0.5 mV, and −70.6 ± 1.0 mV in 10 mM NaCl after photoaging for 2, 4, and 8 days, respectively. Additionally, the hydrodynamic size decreased from the initial value of 159.8 ± 0.7 nm to 156.4 ± 0.9, 153.0 ± 0.8, and 151.7 ± 1.7 nm after photoaging for 2, 4, and 8 days, respectively (Figure 2c). Similarly, TEM results indicated that the particle sizes for PS_0_, PS_2_, PS_4_, and PS_8_ were 159.2 ± 3.0, 155.7 ± 4.2, 152.7 ± 4.5, and 150.4 ± 4.9 nm, respectively. The SEM and TEM images also showed that both pristine and photoaged NPs generally exhibited a regular spherical morphology (Figures S6 and S7), indicating that photoaging occurred primarily at the surface of the NPs without inducing obvious fragmentation.

### NOM Adsorption Modifies Pristine and Photoaged NPs

As observed in the FTIR spectra and corresponding specific wavenumbers (Figure 1b and Table S7), both bulk NOM and NOM fractions exhibited peaks at 1042, 1112, 1388, 1574, 1747, and 3300 cm^–1^, indicating the presence of −OH and C=O groups.^30,53,61,62^ Typically, lower-MW NOM showed more pronounced absorption peaks across all bands, indicating a higher abundance of −OH and C=O groups.^30^ As depicted in the UV–vis spectra (Figure S8), NOM with higher MW contained abundant aromatic components, evidenced by the increased absorbance at 254 nm with increasing MW.^30^ The UV extinction coefficients (SUVA_254_) were as follows: 0.056 L mg^–1^ cm^–1^ for bulk NOM (<0.05 μm), 0.085 L mg^–1^ cm^–1^ for NOM (30 kDa–0.05 μm), 0.071 L mg^–1^ cm^–1^ for NOM (10–30 kDa), 0.065 L mg^–1^ cm^–1^ for NOM (3–10 kDa), and 0.038 L mg^–1^ cm^–1^ for NOM (<3 kDa), respectively. The corresponding aromaticity values were estimated to be 26.0%, 36.6%, 31.6%, 29.2%, and 19.2%, respectively.^63^ Conversely, the lower-MW NOM fractions exhibited stronger fluorescence intensities at *E*_x_ = 300–350 nm/*E*_m_ = 400–500 nm and *E*_x_ = 250–275 nm/*E*_m_ = 380–500 nm (Figure S9). This may be attributed to the higher abundance of aromatic carboxylic and hydroxyl groups in the lower-MW NOM fractions.^35,64,65^ These findings aligned with previous research, indicating that higher-MW NOM fractions contain more aromatic structures, whereas lower-MW fractions are richer in hydrophilic C=O and −OH groups.^66,67^

FTIR analysis revealed that NOM may adsorb onto pristine and photoaged NPs through different interaction mechanisms (Figures 1c,d and S10). For pristine NPs, the adsorption of NOM reduced the intensities of aliphatic segments at 2850 and 2920 cm^–1^ and benzene rings at 1452, 1492, and 3026 cm^–1^ (Figure 1c), indicating NOM likely interacted with these components via hydrophobic and π–π interactions.^52^ Notably, higher-MW NOM appeared to reduce these intensities more substantially (Figure 1c), likely due to its more hydrophobic aromatic structures (Figure S8). Additionally, higher-MW NOM could generate stronger van der Waals forces with NPs,^68,69^ which might be contributing to this phenomenon. The adsorption of NOM also led to the appearance of C=O and −OH functional groups at 1737 and 3280 cm^–1^ on pristine NPs (Figure 1d), likely attributed to the signals from unbonded groups, including −OH and C=O groups of NOM (Figure 1b). Generally, pristine NPs with lower-MW NOM adsorption showed higher intensities for C=O (1737 cm^–1^) and −OH (3280 cm^–1^) groups, likely due to their higher abundance and more pronounced response from these functional groups (Figure 1b). However, for photoaged NPs, the adsorption of NOM reduced the intensities of not only the original aliphatic segments (2850 and 2920 cm^–1^) and benzene rings (1452, 1492, and 3026 cm^–1^) but also the newly generated C=O (1737 cm^–1^) and −OH (3280 cm^–1^) (Figures 1d and S10). This suggests that multiple forces likely contributed to the interaction between NOM and photoaged NPs: the hydrophobic aromatic components of NOM may bind to hydrophobic sites (aliphatic segments at 2850 and 2920 cm^–1^ and benzene rings at 1452, 1492, and 3026 cm^–1^) on photoaged NPs via hydrophobic and π–π interactions, while the oxygen-containing groups (C=O and −OH groups at 1737 and 3280 cm^–1^) on photoaged NPs may interact with the hydrophilic −OH and C=O groups of NOM via hydrogen bonding. Notably, a higher-MW NOM exhibited a greater capacity to reduce the intensities of both hydrophobic and hydrophilic groups on photoaged NPs (Figure 1d). This may be attributed to the stronger van der Waals forces between higher-MW NOM and NPs,^68,69^ which could also enable hydrophobic and π–π interactions, as well as hydrogen bonding. Overall, we hypothesize that the adsorption of NOM on NPs was likely governed by a combination of van der Waals forces, hydrogen bonding, hydrophobic, and π–π interactions, influenced by the NOM MW and the extent of NP photoaging.^16^

The effects of bulk NOM and NOM fractions on the change in zeta potential of pristine and photoaged NPs at 10, 100, and 500 mM NaCl are illustrated in Figures S11, 2a, and S12, respectively. At 100 mM NaCl, there were no visible differences between pristine and photoaged NPs as well as between NPs with and without NOM (Figures 2a and S12). However, at 10 mM NaCl, the surface charges of pristine NPs became more negative in the presence of bulk NOM and NOM fractions (Figure S11). This phenomenon, widely observed for engineered nanoparticles, was attributed to the charge superposition of the adsorbed NOM.^32,66^ However, the presence of NOM reduced the negative charges of photoaged NPs, especially for PS_4_ and PS_8_. While no existing studies have documented this phenomenon, we propose that NOM was likely to interact with these hydrophilic O-containing functional groups on photoaged NPs via hydrogen bonding (Figure 1d), thereby shielding the negative charges or the O-containing functional groups on the surface of the photoaged NPs.

Figures S12 and 2b illustrate the UV_280_ reduction for bulk NOM and NOM fractions postadsorption at 10 and 100 mM NaCl solution, respectively. For pristine NPs, there was a 9.2 ± 1.6% reduction in the UV_280_ of bulk NOM at 10 mM NaCl, indicating NOM sorption onto the NPs (Figure S13). However, the addition of both bulk NOM and NOM fractions did not lead to a visible change in the hydrodynamic size of both pristine and photoaged NPs at 10 mM NaCl (Figure S14). At 100 mM NaCl, the adsorption of bulk NOM on PS_0_, PS_2_, PS_4_, and PS_8_ increased importantly to 63.0 ± 0.8%, 58.0 ± 1.0%, 51.0 ± 0.8%, and 42.2 ± 1.0%, respectively. Meanwhile, there was an important increase in hydrodynamic size from the initial values of 159.8 ± 0.7, 156.4 ± 0.9, 153.0 ± 0.8, and 151.1 ± 1.7 nm for PS_0_, PS_2_, PS_4_, and PS_8_ to 165.4 ± 1.4, 164.8 ± 1.9, 161.4 ± 1.7, and 158.3 ± 1.8 nm, respectively, when exposed to bulk NOM (Figure 2c). This was attributed to NOM adsorption rather than NOM aggregation, as no NOM aggregates were detected by DLS at 100 mM NaCl (Figure S15). The increased adsorption of bulk NOM at higher salt concentrations can be attributed to the compaction of the electrostatic layer between the NPs and NOM. Notably, at 100 mM NaCl, the adsorption at UV_280_ decreased with the aging of NPs. Similar trends were observed across various NOM fractions, suggesting that photochemical weathering reduced the adsorption of NOM across all fractions. Generally, the NOM with relatively high MW exhibited relatively high adsorption on both PS_0_ and PS_2_. However, for the more aged PS_4_ and PS_8_, the adsorption of NOM (>30 kDa) was not the highest compared to lower-MW NOM fractions. Figure 2d shows the adsorption layer thickness determined by comparing the hydrodynamic sizes of the NPs before and after NOM adsorption. Generally, the mean adsorption layer thickness of NOM fractions increased with their MW, indicating that higher-MW fractions formed thicker layers on both pristine and photoaged NPs. Notably, although bulk NOM showed higher adsorption on the NPs compared to NOM > 30 kDa, the mean adsorption layer thickness of bulk NOM was generally lower than that of NOM > 30 kDa. Thus, there was no consistent correlation between the mean adsorption layer thickness and adsorption capacity. The surface coverage of NOM on NPs was estimated based on the adsorption amount and assumed NOM size (Table S4). The results indicated that surface coverage of NOM decreased with the photoaging of NPs, and higher-MW NOM exhibited lower surface coverage, potentially due to spatial constraints between NOM molecules.^70^

### Photochemical Weathering Stabilizes NPs

The effect of photochemical weathering on the aggregation tendency of NPs was examined in monovalent solutions (Figure S16). As ionic strength increased, the attachment efficiency of both pristine and photoaged NPs increased, due to the charge screening effect of Na^+^, which reduced the electrostatic repulsion between the NPs.^9^ The CCC value of the pristine NPs was measured at 550 mM NaCl, indicating their high stability. However, for photoaged NPs, the CCC values exceeded 1000 mM, indicating that photoaged NPs were much more stable than pristine ones. At equivalent NaCl concentrations, the attachment efficiencies decreased with prolonged photoaging, indicating a reduction in NP stability as the extent of aging increased. Notably, the zeta potential of pristine NPs became increasingly negative with prolonged photoaging, consequently intensifying the electrostatic repulsion among the photoaged NPs. Therefore, it can be inferred that electrostatic interaction played a pivotal role in stabilizing NPs following photoaging, in line with the previous studies.^9^ The DLVO theory predicted high energy barriers (>20 *k*_B_*T*) for both pristine and photoaged NPs at 100 mM NaCl. However, at 500 mM NaCl, no energy barrier was observed for PS_0_, while energy barriers of 12.5 *k*_B_*T*, 28.4 *k*_B_*T*, and 31.5 *k*_B_*T* were noted for PS_2_, PS_4_, and PS_8_, respectively (Figure S17). One study also suggested that the stabilization of NPs may be induced by the release of dissolved organic molecules due to steric repulsion.^9^ However, in this study, both pristine and photoaged NPs underwent a washing step to remove dissolved organic molecules, and thus, these molecules were not responsible for the enhanced stability observed in our findings.

### NOM Stabilizes Pristine NPs but Destabilizes Photoaged NPs

**Figure 3:**
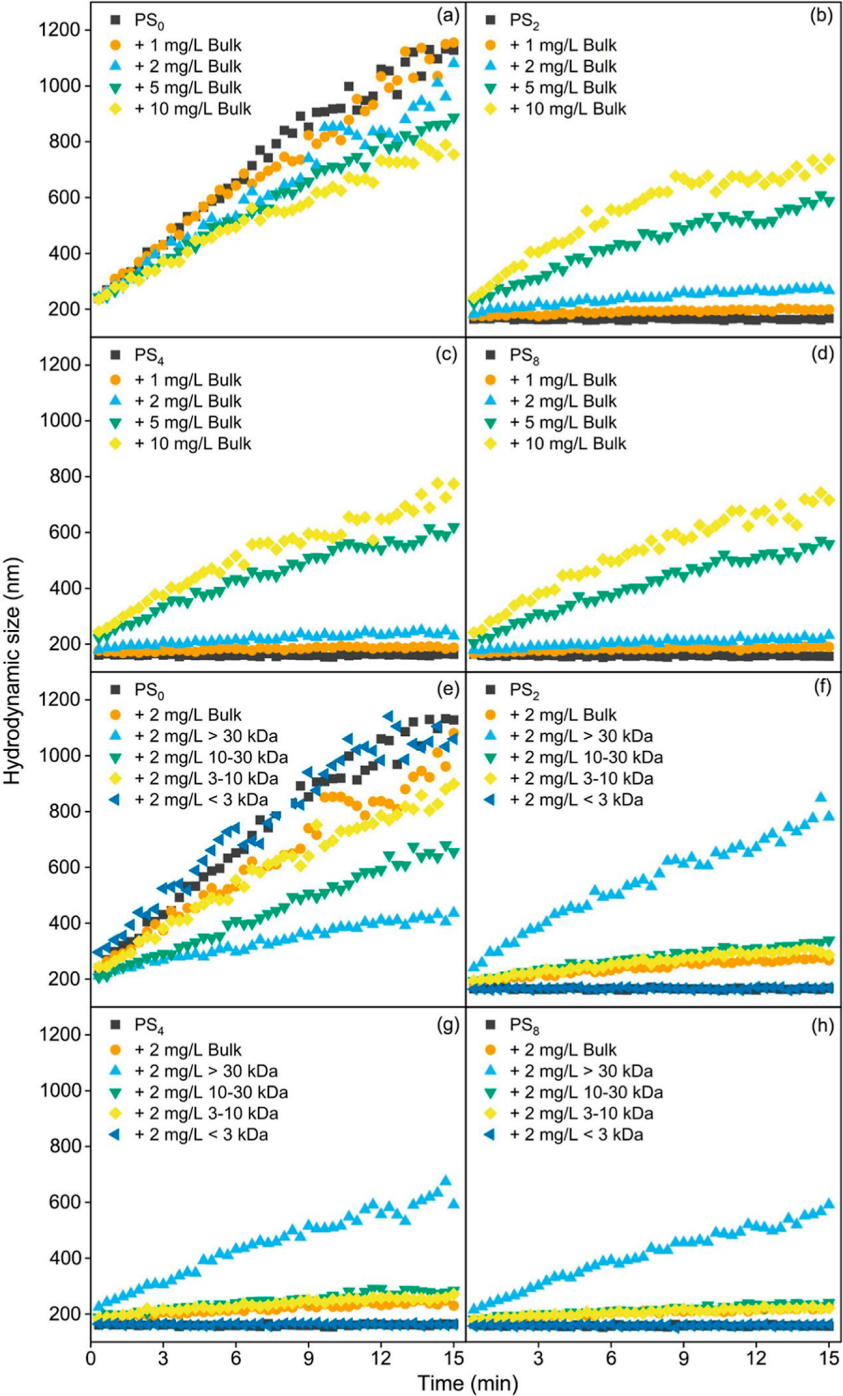
Effect of bulk NOM concentration (1, 2, 5, and 10 mg/L) and different MW NOM fractions (2 mg/L) on the hydrodynamic size (intensity-weighted) change of pristine and photoaged NPs at 500 mM NaCl. Subscripts 0, 2, 4, and 8 mean aging times of 0, 2, 4, and 8 d.

**Figure 4:**
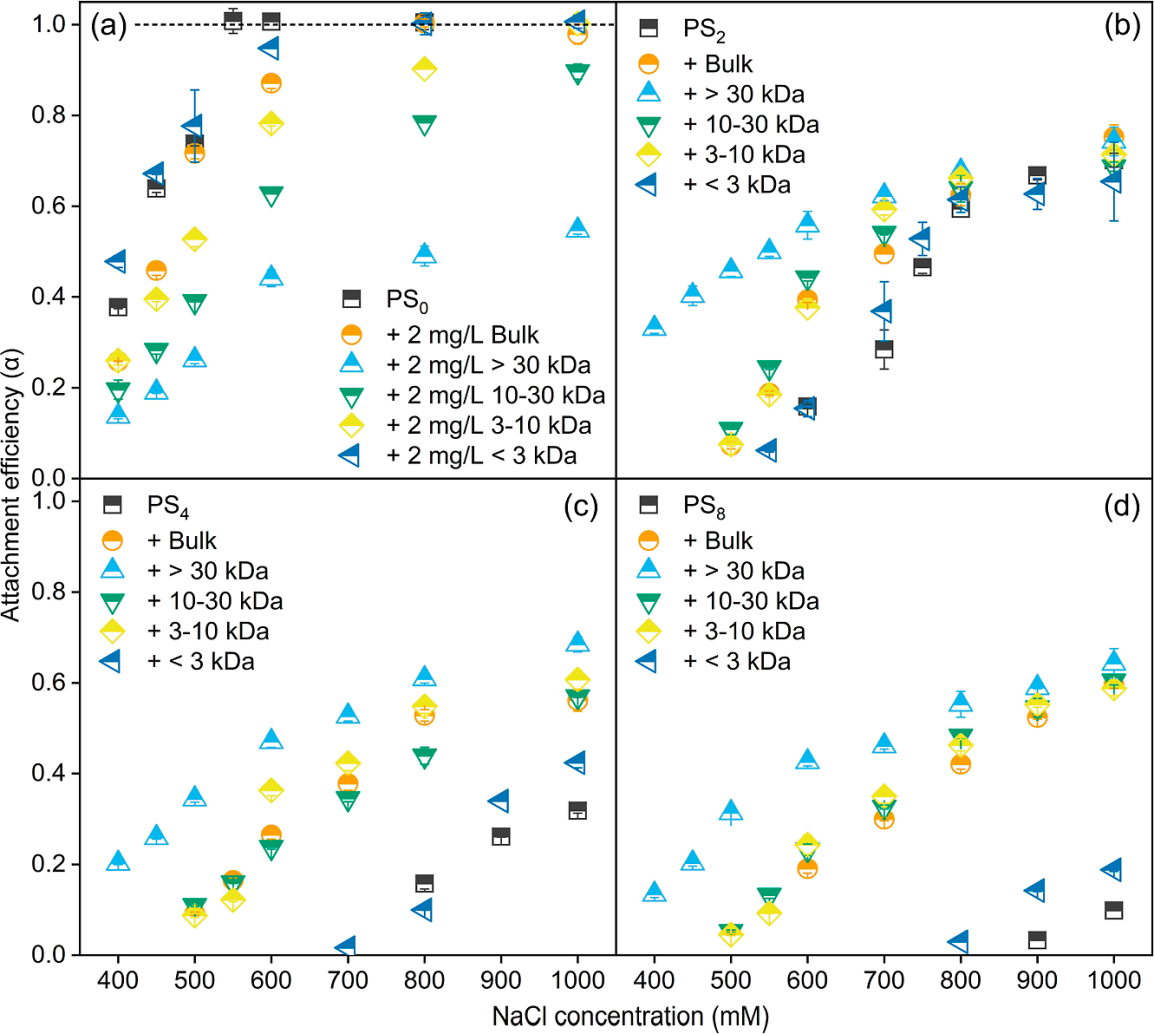
Aggregation kinetics of pristine and photoaged NPs without and with NOM (2 mg/L) in NaCl solutions. Subscripts 0, 2, 4, and 8 mean aging times of 0, 2, 4, and 8 d. Error bars represent the mean ± SD (*n* = 2 or 3).

The effect of bulk NOM on the hydrodynamic size change of pristine and photoaged NPs in 500 mM NaCl is shown in Figures 3a–d and S18–S21. The presence of bulk NOM slowed the rate of increase in the hydrodynamic size of pristine NPs (Figure 3a). The Litesizer measurements indicated that the intensity, volume, and number-weighted hydrodynamic sizes all decreased in the presence of bulk NOM (Figures S18 and S19), suggesting that bulk NOM hindered the aggregation of pristine NPs under this condition. Additionally, the inhibitory effect of bulk NOM on NP aggregation increased with rising NOM concentrations (Figure 3a). Figure 4a further illustrates the change in the attachment efficiency with varying NaCl concentrations. As observed, in the presence of bulk NOM, the CCC value of pristine NPs increased from 550 mM to around 630 mM NaCl, suggesting that NOM enhanced the stability of NPs in monovalent solutions. The electrostatic interaction had a negligible effect, as evidenced by no important difference in the zeta potentials of pristine NPs with and without NOM (Figure S13). We hypothesize the electro-steric repulsion induced by the adsorption layer might be a primary factor stabilizing the pristine NPs.^37^ As reported, the adsorption of the NOM layer can disrupt the original ionic diffuse layer of particles, leading to an expansion of the ionic diffuse layer, which may enhance the stability of NPs.^71^ Additionally, based on the theoretical interaction energy profiles at 500 mM NaCl, a high energy barrier was present between pristine NPs in the presence of NOM (Figure S22a). This barrier resulted from the additional long-range steric repulsion that occurred when the separation distance decreased to less than twice the thickness of the NOM adsorption layer. The stabilization of NPs and engineered nanoparticles by NOM via steric repulsion has also been extensively documented in previous studies.^11,39,44^

Nevertheless, the addition of NOM increased the hydrodynamic size of photoaged NPs (Figure 3b–d). This phenomenon was not due to NOM aggregation, as DLS did not detect NOM aggregates (Figure S15). The intensity, volume, and number-weighted size distributions of photoaged NPs with and without NOM over time were examined at 500 mM NaCl (Figures S20 and S21). All three types of size distributions generally exhibited uniform single peaks with minimal variation from each other and showed similar trends over time, indicating that the majority of photoaged NPs aggregated in the presence of NOM. The attachment efficiencies of photoaged NPs at all tested NaCl concentrations also increased after the addition of NOM (Figure 4b–d), suggesting that NOM destabilized photoaged NPs. Notably, the aggregation of photoaged NPs occurred under relatively high NaCl concentrations, where NOM had minimal effect on the zeta potential of NPs (Figure S13), suggesting that other forces rather than electrostatic interaction were at play. It has been reported that in addition to steric repulsion, soluble polymers can induce destabilization of colloids/nanoparticles through depletion attraction^22,25^ or polymer bridging.^21,72^ As NOM was adsorbed on photoaged NPs (Figure 2b), it is unlikely that depletion attraction was the mechanism responsible for the destabilization of photoaged NPs by NOM. In this study, we hypothesize that NOM might serve as a bridging agent among the photoaged NPs, thereby promoting their aggregation. The XDLVO theory, which considers steric attractive interactions (i.e., polymer bridging), accurately predicted this destabilization phenomenon (Figure S22b–d). The long-range polymer bridging created a deep primary energy minimum as the separation distance decreased to less than the thickness of the NOM layer. At relatively low salt concentrations (e.g., 100 mM), the strong electrostatic repulsion created a substantial energy barrier between the photoaged NPs, preventing them from coming close enough for polymer bridging to occur (Figure S22b1–d1). However, at higher salt concentration (e.g., 500 mM), the electrical double layer was compressed, allowing the NPs to approach each other and enabling polymer bridging to take effect (Figure S22b2–d2). Few studies have reported polymer bridging by NOM in environmental colloid chemistry.^27,28^ Nason et al. reported that Pony Lake fulvic acid (PLFA, > 2 mg/L) destabilized citrate-stabilized Au nanoparticles in 80 mM KCl, which was explained by bridging flocculation driven by hydrophobic interactions between adsorbed PLFA molecules on adjacent Au nanoparticles.^28^ Pradel et al. demonstrated that sodium alginate a model polysaccharide could bridge pristine PS NPs by binding multiple particles together, while the resulting clusters were further stabilized through steric repulsion.^27^

As reported, steric stabilization occurs with high adsorbed amounts, but polymer bridging requires sufficient unoccupied surface area on particles for the attachment of polymer chain segments from other particles.^72^ Typically, an optimal polymer dosage corresponds to low adsorbed amounts, and an excess of polymer can lead to restabilization.^72^ In this study, although photoaging reduced the amount of NOM adsorbed and the surface coverage on NPs (Table S4), low-MW NOM fractions (e.g., < 10 kDa) at 2 mg/L still exceeded 100% surface coverage on photoaged NPs. Furthermore, the destabilization capacity of the NOM on photoaged NPs increased with the concentration of the NOM (Figure 3b–d). It suggests that the adsorbed amount or surface coverage did not solely determine the distinct role of NOM in the stability of pristine and photoaged NPs. We propose that the unique adsorption configurations of NOM on pristine and photoaged NPs might play a crucial role.

**Figure 5:**
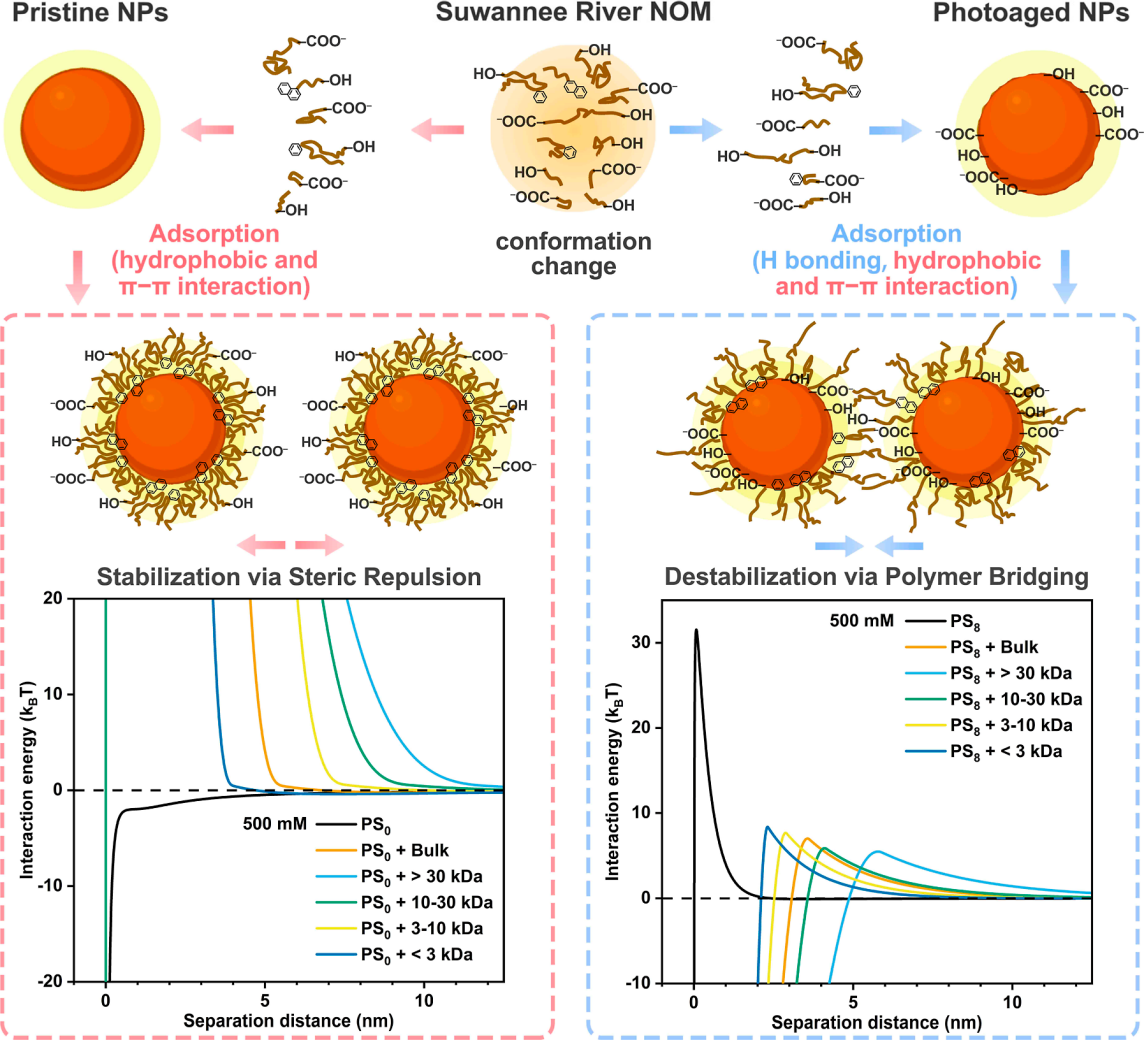
Schematic diagram illustrating the mechanisms of NOM adsorption and the stabilization or destabilization of NPs by NOM, as well as the DLVO and steric model interaction energy curves at 500 mM NaCl.

It is commonly believed that NOM in solution has a micelle-like structure, with hydrophilic moieties exposed on the external part and the hydrophobic domain hidden in the inner part.^73−75^ Conformational rearrangements could occur, enabling favorable moieties to adsorb onto solid surfaces.^75−77^ The hydrophobic components of NOM might tend to bind with the surfaces of pristine NPs through hydrophobic and π–π interactions (Figure 1c), while hydrophilic segments might extend into the surrounding solution (Figure 5).^60,78^ These hydrophilic segments might inhibit NP aggregation through steric repulsion. Compared with pristine NPs, the surface of photoaged NPs was more heterogeneous, containing both aromatic segments and O-containing functional groups (Figure 1a). This surface heterogeneity may allow the NOM to adsorb through both hydrophobic and hydrophilic interactions (Figure 1d). The hydrophobic and π–π interactions likely facilitate the adsorption of the hydrophobic components of NOM onto the nonoxidized regions of the photoaged NPs, while hydrogen bonding may occur between the hydrophilic segments of NOM and the oxidized regions on the photoaged NPs (Figure 5). Additionally, the remaining hydrophobic or hydrophilic segments of NOM in solution may bind with the heterogeneous regions on other photoaged NPs, effectively bridging two or more photoaged NPs. In previous studies, the presence of HA stabilized both pristine and photoaged PS NPs in monovalent solutions, but the stabilization capacity was weakened on photoaged NPs due to less HA adsorption.^29,37^ The distinct effect of NOM on photoaged NPs observed in this study compared with previous studies may be attributed to differences in aging extent. Earlier studies likely involved less photoaged NPs, with aging times of 1 day or less, and CCC values that were determinable and below 1000 mM.^37,41^ HA adsorption on these photoaged NPs was likely dominated by hydrophobic and π–π interactions with minimal hydrogen bonding. In contrast, the increased oxidation in this study may reduce these hydrophobic interactions while enhancing the hydrogen bonding between NOM and photoaged NPs. Thus, we propose that sufficient surface hydrogen bond donors (O-containing functional groups) on photoaged NPs are essential for the formation of a polymer bridging.

### MW-Dependent Stabilization and Destabilization Capacity

NOM, being a heterogeneous mixture of components with varying MW and chemical properties, exhibits chemical heterogeneity among its fractions.^79^ This molecular heterogeneity plays a crucial role in determining the formation of an eco-corona, which subsequently impacts the stability of nanoparticles.^36,44^ The impact of MW-fractioned NOM on the hydrodynamic size change of pristine NPs in a 500 mM NaCl solution is depicted in Figure 3e–h. Compared to pristine NPs in the absence of NOM fractions, NOM with relatively large MWs (i.e., > 30 kDa, 10–30 kDa, and 3–10 kDa) notably inhibited the increase in the hydrodynamic size of NPs, while NOM with MW below 3 kDa seemed to promote the increase in hydrodynamic size in the initial stage. At all tested NaCl concentrations, NOM with relatively large MWs (i.e., > 30 kDa, 10–30 kDa, and 3–10 kDa) inhibited the aggregation of pristine NPs (Figure 4b–d). Notably, although NOM < 3 kDa exhibited some destabilization on pristine NPs under relatively low NaCl concentrations (≤500 mM), the CCC value of pristine NPs with NOM < 3 kDa was higher than that of pristine NPs alone (610 mM vs 550 mM). Generally, the stabilization capacity of NOM fractions increased with the increase in MW. Due to higher aromaticity and fewer carbonyl groups, NOM with higher MW exhibited higher adsorption capacity on pristine NPs (Figure 2b). Additionally, the larger size of the high-MW NOM contributed to the formation of a thicker adsorption layer on the NPs (Figure 2d). According to the steric model (Figures 5 and S22a), a higher-MW NOM could generate longer-range steric repulsion, producing a high energy barrier at a larger separation distance, thus demonstrating stronger stabilization capacity.

For photoaged NPs, NOM < 3 kDa did not induce obvious changes in the hydrodynamic size at 500 mM NaCl, whereas NOM with larger MWs, particularly NOM > 30 kDa, caused an increase in the hydrodynamic size of PS_2_, PS_4_, and PS_8_ (Figure 3f–h). The attachment efficiencies of photoaged NPs in the presence of NOM fractions were further investigated across a wide range of NaCl concentrations. Generally, similar to bulk NOM, all of the NOM fractions destabilized the photoaged NPs. Notably, the destabilization capacity of the NOM fractions increased with increasing MW. NOM with relatively large MW generally formed a thicker adsorption layer on photoaged NPs (Figure 2d). Based on the theoretical calculations of the steric model, the adsorption layer thickness largely determined the separation distance at which polymer bridging occurs (Figures 5 and S22b–d). NOM with a relatively large MW could serve as a “large bridge”, binding photoaged NPs at a relatively long distance (Figure 5). Thus, the destabilization of photoaged NPs was strongly dependent on the MW of NOM.

## Environmental Implications

The aggregation behavior and colloidal stability of NPs are highly impacted by natural weathering processes, such as photochemical weathering,^9^ and eco-corona formation.^11,39^ This study revealed that the role of photochemical weathering and eco-corona in the colloidal stability of NPs was more complicated than what we previously expected.^37^ Although both photoaging and NOM adsorption are known to stabilize NPs in monovalent electrolyte solutions,^9,11,39^ our study highlighted that NOM stabilized pristine NPs most likely via steric repulsion but destabilized photoaged NPs via polymer bridging in monovalent electrolyte solutions. The modification of NPs through photochemical weathering likely impacts eco-corona formation.^80,81^ Although photoaging reduces the amount of eco-corona on NPs,^36^ this is not the primary reason for the destabilization of photoaged NPs. We propose that distinct interaction between NOM and pristine/photoaged NPs induced distinct eco-corona conformation on pristine and photoaged NPs, thus leading to distinct stability effects. In monovalent solutions, NOM typically stabilizes NPs or engineered nanoparticles via steric repulsion,^11,39,79^ while the destabilization phenomenon of NOM usually occurs in the presence of multivalent cations (e.g., calcium) via cation bridging.^32,82^ This study highlighted the destabilization role of NOM in monovalent solutions in environmental colloid chemistry, providing new insights into the stability and fate of NPs and engineered nanoparticles under complex aquatic conditions. NOM is diverse in terms of molecular size and properties.^79^ NOM with relatively high MWs typically exhibits relatively high aromaticity and hydrophobicity but low hydrophilicity.^44^ In this study, higher-MW NOM fractions displayed greater adsorption on both pristine and photoaged NPs, indicating that the photoaging did not notably change the adsorption preference of NPs based on the NOM size. In addition, despite their lower abundance, NOM with relatively high MWs played a dominant role in either stabilizing pristine NPs or destabilizing photoaged NPs. This highlights the importance of considering the heterogeneity in the molecular size distribution of environmental NOM when interpreting its effects on the transport and fate of NPs.

It should be noted that commercial PS NPs used in this study are highly stable, making it challenging to observe aggregation behaviors under typical freshwater conditions. To address this, high salinity conditions were chosen to better observe and understand the interactions and aggregation behaviors of the NPs. Thus, the findings are relevant to estuarine or marine ecosystems, where elevated salinity levels are common. However, the underlying mechanisms observed in this study, such as the interactions of NOM with pristine and weathered NPs and the resulting aggregation processes, are fundamental and may also apply to less stable NPs or scenarios in freshwater systems. This suggests that while specific outcomes may vary, the broader insights provided by this research could contribute to understanding NP behavior across a range of aquatic environments.

## Supporting Information Available

Evaluation of the accelerating factor; spectrum of the mercury lamp for accelerated photoaging; UV289 change of PS NPs under mercury lamp exposure; pictures showing the color change of NPs after photoaging; UV289, TOC, and size of pristine and photoaged NPs; UV absorbance of bulk NOM before and after adsorption at 100 mM NaCl; UV absorbance of the filtrates of washed pristine and photoaged NPs; aggregation kinetics measurements; detailed characterization; DLVO and steric model; parameters for the calculations of steric interaction; functional groups of pristine and photoaged PS NPs at their respective wavenumbers; TEM images of pristine and photoaged NPs; SEM images of pristine and photoaged NPs; UV absorbance of bulk NOM and different NOM fractions; 3D-EEM spectra of bulk NOM and different NOM fractions; reduction in UV_280_ of bulk NOM and NOM fractions after adsorption on pristine and photoaged NPs in 10 mM NaCl; functional groups of bulk NOM and NOM fractions at their respective wavenumbers; zeta potential of pristine and photoaged NPs with and without bulk NOM and NOM fractions in 10 mM NaCl; hydrodynamic size of pristine and photoaged NPs with and without bulk NOM and NOM fractions in 10 mM NaCl; FTIR spectra of PS_2_ and PS_4_ before and after adsorption with bulk NOM in 100 mM NaCl; reduction in UV280 (NOM removal) of bulk NOM and NOM fractions after adsorption on pristine and photoaged NPs in 10 mM NaCl; zeta potential of pristine and photoaged NPs with and without bulk NOM and NOM fractions in 10 mM NaCl; zeta potential of pristine and photoaged NPs with and without bulk NOM and NOM fractions in 500 mM NaCl; hydrodynamic size of pristine and photoaged NPs with and without bulk NOM and NOM fractions in 10 mM NaCl; size characterization results of bulk NOM (10 mg/L) over time at 100 and 500 mM NaCl; aggregation kinetics of pristine and photoaged NPs without NOM and with bulk NOM; DLVO interaction energy of pristine and photoaged NPs at 100 and 500 mM NaCl solutions; intensity, volume, and number-weighted size distributions of pristine NPs with and without bulk NOM and NOM > 30 kDa at 500 mM over time; and DLVO and steric interaction energy of pristine or photoaged NPs in the absence and presence of bulk NOM and NOM fractions at 100 and 500 mM NaCl solutions (PDF)
