# Supplementary material for: Natural Organic Matter Stabilizes Pristine Nanoplastics but Destabilizes Photochemical Weathered Nanoplastics in Monovalent Electrolyte Solutions
Source: Environ Sci Technol. 2025 Jan 15;59(3):1822–34. doi: 10.1021/acs.est.4c11540 (PMC11780734; doi:10.1021/acs.est.4c11540)
Supplement: Supplementary file 1 — es4c11540_si_001.pdf [file es4c11540_si_001.pdf]

Supporting Information for:

Natural Organic Matter Stabilizes Pristine Nanoplastics but Destabilizes Photochemical Weathered Nanoplastics in Monovalent Electrolyte Solutions

Yanghui Xu [a,b], Xintu Wang [a], Jan Peter van der Hoek [b,c], Gang Liu [a,b,d,*], Kim Maren Lompe [b]

[a.] *Key Laboratory of Drinking Water Science and Technology, Research Centre for Eco-Environmental Sciences, Chinese Academy of Sciences, Beijing, 100085, P. R. China*

[b.] *Section of Sanitary Engineering, Department of Water Management, Faculty of Civil Engineering and Geosciences, Delft University of Technology, Stevinweg 1, 2628 CN Delft, the Netherlands*

[c.] *Waternet, Department Research & Innovation, P.O. Box 94370, 1090 GJ Amsterdam, the Netherlands*

[d.] *University of Chinese Academy of Sciences, Beijing, 100049, China*

Corresponding author:

Gang Liu,

Research Center for Eco-Environmental Sciences, Chinese Academy of Sciences

Sanitary Engineering, CiTG, Delft University of Technology

g.liu-1@tudelft.nl; gliu@rcees.ac.cn

The supplementary material includes 4 texts, 22 figures and 7 tables in 32 Pages.

Text S1. Evaluation of the Accelerating Factor. A mercury lamp (500 W) emitting UV light with an intensity of approximately 35 mW/cm^2 was employed to age PS NPs. The spectrum of the mercury lamp is shown in Figure S1. was evaluated by comparing the photoaging of PS NPs under actual sunlight exposure in the Netherlands. PS NPs at a concentration of 20 mg/L were initially photoaged for durations of 3, 6, 12, and 24 hours using a mercury lamp. The extent of aging was determined by measuring the UV absorbance at 289 nm with UV-vis absorbance spectroscopy. As depicted in Figure S2, the UV289 initially increased slightly and then decreased with prolonged photoaging. Real sunlight exposure was conducted on a house roof for five days in the Netherlands during the summer of 2023. Based on calculations, five days of sunlight exposure equated to six hours of mercury lamp aging. Thus, one day of mercury lamp aging corresponded approximately to 20 days of sunlight exposure in the Netherlands.

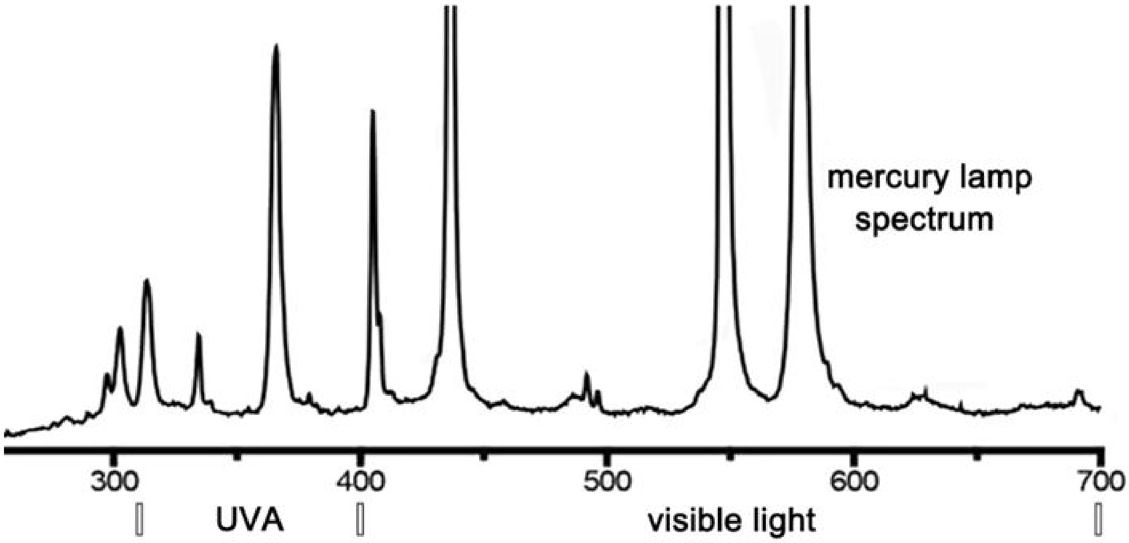

Figure S1. The spectrum of the mercury lamp for accelerated photoaging

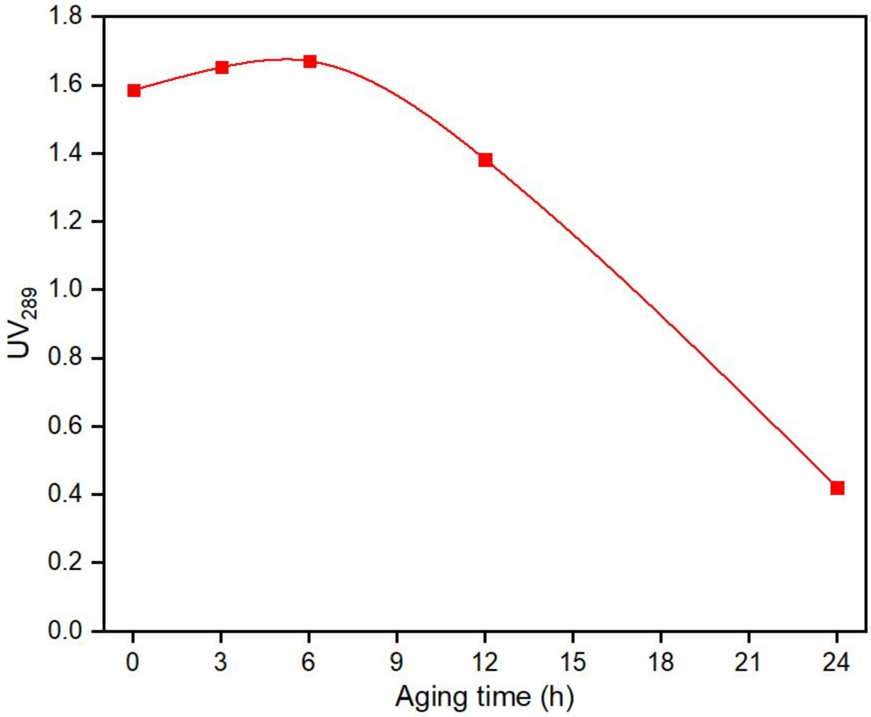

Figure S2. The change in UV absorbance at wavelength 289 nm of PS NPs (20 mg/L) under mercury lamp exposure.

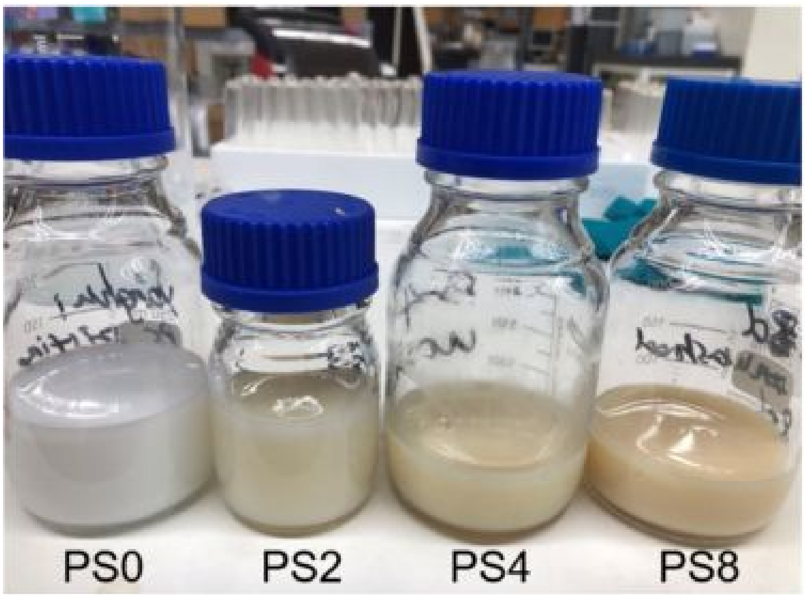

Figure S3. Pictures showing the color change of NPs after photoaging

Table S1. The UV289 (measured at approximately 4.68×10^{12} particles/L), TOC (measured at approximately 4.68×10^{12} particles/L) and size (measured with TEM) of PS_0, PS_2, PS_4 and PS_8.

	PS_0	PS_2	PS_4	PS_8
UV289	0.967	0.927	0.916	0.902
TOC (mg C/L)	29.2 ± 0.1	27.8 ± 0.2	24.7 ± 0.1	23.6 ± 0.3
Size (nm)	159.2 ± 3.0	155.7 ± 4.2	152.7 ± 4.5	150.4 ± 4.9

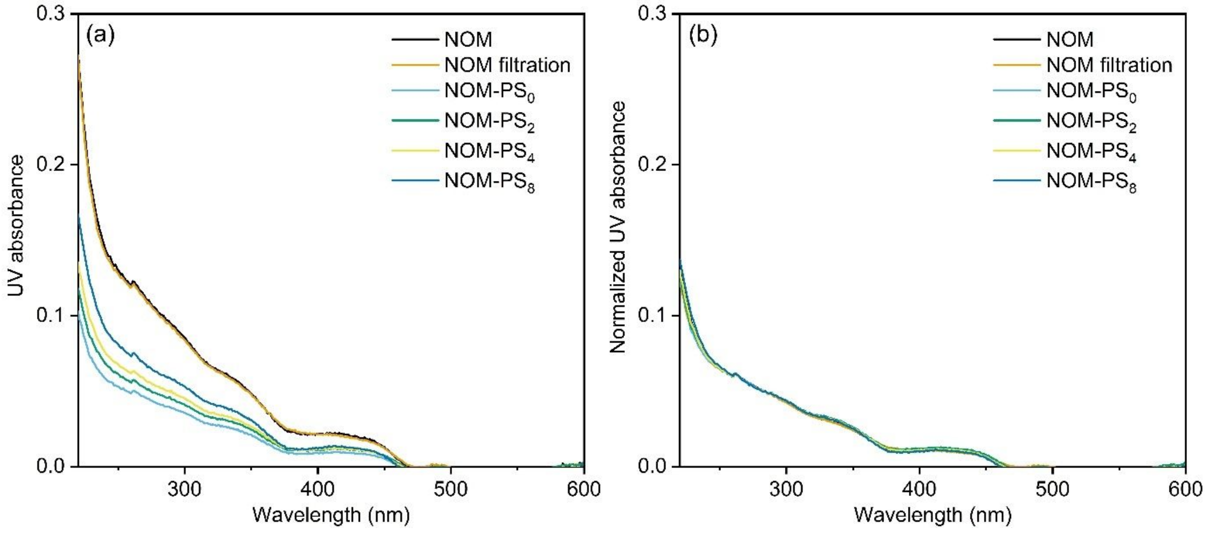

Figure S4. (a) UV absorbance of bulk NOM (2 mg C/L) before and after adsorption by pristine and photoaged NPs at 100 mM NaCl. (b) corresponding normalized UV absorbance (SUVA) to NOM mass that was estimated using UV_{280}.

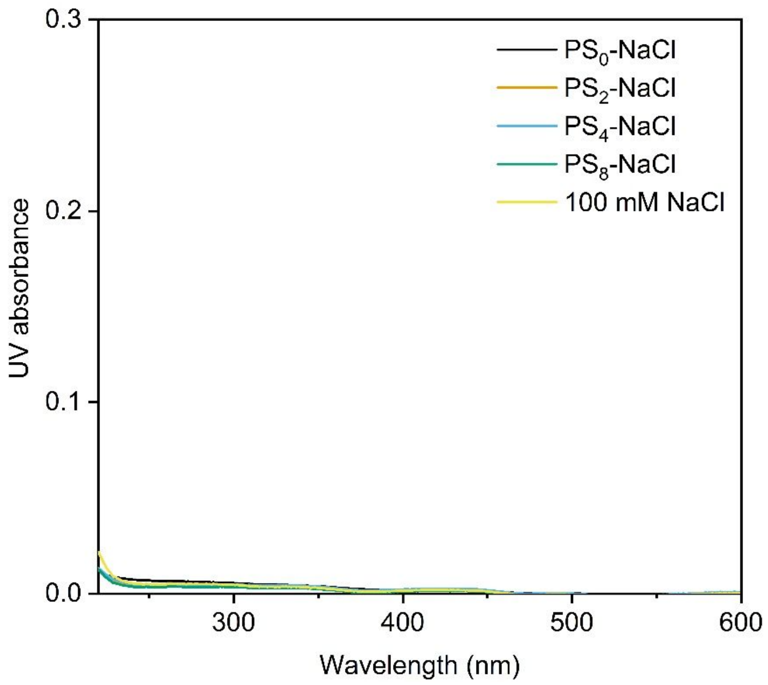

Figure S5. UV absorbance of the filtrates of washed pristine and photoaged NPs (10 mg/L in 100 mM NaCl) after 0.1 μm filtration, as well as 100 mM NaCl (blank).

Text S2. Aggregation Kinetics Measurements. Time-resolved dynamic light scattering (DLS) technique was used to examine the aggregation kinetics of NPs in NaCl solutions utilizing a Malvern Zetasizer instrument (Nano ZS, Malvern, UK). The instrument featured a He–Ne laser operating at a wavelength of 633 nm and maintained a fixed scattering angle of 173°.[1] The NOM and NP samples were pre-mixed in ultrapure water, and the pH was adjusted to 6.0 ± 0.1 using 0.1 M HCl and NaOH. The concentration of NPs was 10 mg/L, a common concentration for aggregation kinetic studies.[2-5] Bulk NOM was adjusted to concentrations ranging from 1 to 10 mg C/L and NOM fractions to 2 mg C/L, falling within the typical range of NOM concentrations found in natural waters.[3, 6, 7] Before measurement, 0.5 mL of NPs or NPs/NOM were added to a 1-cm quartz cuvette, followed by the addition of 0.5 mL of NaCl solution with the desired concentration (ranging from 200 mM to 2000 mM). The mixture was immediately measured, and the aggregation kinetic was determined by continuously monitoring the hydrodynamic size change of samples every 30 seconds over a 15-minute period.[8] Duplicates or triplicates were conducted for each sample.

The aggregation kinetics of PS NPs in the early stages were assessed by measuring the initial rate of hydrodynamic size (Dh) change over time (t). The initial aggregation rate constant (k) was directly proportional to the initial rate of Dh increase and was normalized to the initial concentration of PS NPs in the suspension (M0) (Equation (S1)).[6]

$$k \propto \frac{\left(\frac{dD_h(t)}{dt}\right)_{t\to 0}}{M_0}, \tag{S1}$$

The initial rate of the linear increase in Dh for PS NPs with time (dDh(t)/dt) was determined from t = 0 until Dh reached 1.5Dh0. This value was calculated using linear least squares regression analysis. The attachment efficiency (α), used to characterize the stability of PS NPs, was calculated by normalizing k under various solution conditions to kf under rapid aggregation conditions.[9, 10]

$$\alpha = \frac{k}{k_f} = \frac{\left(\frac{dD_h(t)}{dt}\right)_{t\to 0}}{\left(\frac{dD_h(t)}{dt}\right)_{t\to 0, fast}}, \tag{S2}$$

Two distinct regimes, termed reaction-limited and diffusion-limited aggregation, can be clearly identified. In the initial reaction-limited stage ($\alpha < 1$), the aggregation efficiency of PS NPs increased with increasing salt concentration, consistent with DLVO theory.[9, 11] The salt concentration at which attachment efficiency (α) reaches its maximum value 1 was termed the critical coagulation concentration (CCC), serving as an indicator of the aggregation potential of PS NPs.[9, 12] In this study, the CCC values of most samples, such as pristine and photoaged NPs in the presence of NOM, were not determined due to their strong stability.

The Zetasizer typically reports the intensity-weighted hydrodynamic size of nanoparticles, which may overestimate larger particles due to their stronger light scattering.[13] To ensure that the observed aggregation of NPs analyzed using the Zetasizer was not biased by a few larger aggregates, the additional aggregation measurements using the Litesizer DLS 700 (Anton Paar, Austria) were conducted to detect the changes in the intensity, volume, and number-weighted hydrodynamic sizes. The aggregation processes of pristine (PS_0) and photoaged (PS_4) NPs (10 mg/L) in the absence and presence of bulk NOM (10 mg/L) in 500 mM NaCl were studied.

Text S3. Characterization. The UV-vis spectroscopy (G10S UV-Vis, Thermo Fisher Scientific) and three-dimensional excitation-emission matrix (3D-EEM) fluorescence spectroscopy (Horiba Scientific) were employed to characterize the chemical properties of bulk NOM and NOM fractions. The hydrodynamic size and zeta potential of NPs in the absence and presence of NOM were measured using the Zetasizer Nano ZS90 (Malvern Instruments, UK). Before measurement, pristine or photoaged NPs (10 mg/L) and NOM or NOM fractions (2 mg C/L) were mixed in 10 and 100 mM NaCl for 24 h (preliminary tests indicated that no aggregation of NPs or NOM occurred at these conditions). The average adsorption layer thickness of NOM on the NPs was calculated based on the differences in the hydrodynamic size of NPs before and after interaction with NOM.

The size and morphology of pristine/photoaged NPs in ultrapure water were examined using a scanning electron microscope (SEM, Quattro, FEI)) and a transmission electron microscope (TEM, Tecnai G20, FEI Corp, USA). For SEM measurement, NPs were pre-filtered onto 0.1 μm PES membranes, dried at 40 °C and then coated with gold. Prior to TEM analysis, the samples were dropped onto 200 mesh carbon-coated copper grids and dried at 40 °C.

Attenuated total reflectance Fourier transform infrared spectroscopy (ATR-FTIR, Nicolet iN10, Thermo Fisher Scientific) was used to detect the surface functional groups of NPs and NOM, as well as their interactions utilizing wavenumbers ranging from 400 to 4000 cm^{-1}.[9] The NP samples were prepared by drying concentrated NPs on a glass slide at 40 °C to avoid changes of NPs, while NOM samples were freeze-dried using a freeze-dry instrument (BK-FD12PT, Vertical Freeze Dryer, BIOBASE BIODUSTRY) before FTIR analysis. To detect the interactions between NPs and NOM, 100 mL reaction solutions containing 10 mg/L pristine or photoaged NPs and 2 mg/L bulk NOM or NOM fractions were prepared and incubated for 24 h in 100 mM NaCl. The mixture was then filtered using 0.1 μm PES membranes, and the samples on the membranes were dried at 40 °C before being analyzed using FTIR. Twenty

discrete spectral measurements were conducted linearly and subsequently integrated to synthesize a composite spectrum.

Text S4. DLVO and Steric Model. The classical Derjaguin-Landau-Verwey-Overbeek (DLVO) theory includes van der Waals and electrostatic double layer interactions.[1, 14] The total interaction energy (E_{DLVO}) defined as the sum of the van der Waals interaction energy (E_{vdW}) and electric double layer interaction energy (E_{EDL}). In the presence of NOM, the extend DLVO (XDLVO) considering a steric (polymer-mediated) model was used to investigate interaction energies between NPs based on repulsive steric interaction and attractive polymer bridging interaction.

DLVO. The equations of E_{DLVO}, E_{vdW} and E_{EDL} are given as follows:

$$E_{DLVO} = E_{vdW} + E_{\mathrm{EDL}} \tag{S3}$$

$$E_{vdW} = -\frac{A_{121}R}{12h(1+\frac{14h}{\lambda_0})} \tag{S4}$$

$$E_{\mathrm{EDL}} = 32\pi\varepsilon_0\varepsilon_r R(k_BT/ze)^2 tanh\,(\frac{\Psi}{4k_BT/ze})^2 exp(-\kappa h) \tag{S5}$$

where A_{121} (J) is the Hamaker constant of NP-water-NP system. $A_{121} \approx ((A_{11})^{1/2} - (A_{22})^{1/2})^2$, where A_{11} and A_{22} denote the Hamaker constants of NPs and water ($A_{22} \approx 3.7 \times 10^{-20}$ J) in a vacuum. The Hamaker constants of PS_0, PS_2, PS_4, and PS_8 were estimated to be 5.1×10^{-21} J, 1.2×10^{-21} J, 5.0×10^{-22} J, and 3.5×10^{-22} J based on fitting results.[2, 15] R (nm) is the particle radius. h (nm) is the separation distance. ε_0 and ε_r are the permittivity of the vacuum (8.854×10^{-12} C/(V□m)) and the relative dielectric constant of the liquid (78.5), receptively. k_B is the Boltzman constant (1.381×10^{-23} J/K). T is absolute temperature (298 K), z is the valence state of ion. e is the electron charge (1.602×10^{-19} C). Ψ (V) is the potential of NPs. κ is the inverse Debye length (Eq. 6).

$$\kappa^{-1} = \sqrt{\frac{\varepsilon_r\varepsilon_0 k_BT}{2N_A I e^2}} \tag{S6}$$

where NA is the Avogadro constant (6.02×10^{23} mol^{-1}), and I is the ionic strength of the solutions, $I=0.5\sum c_i z_i^2$, ci is the molarity of ion. The Debye length or ionic diffuse layer is a measure of

the range of electrostatic interactions. k^{-1} is calculated as 0.96 nm and 0.43 nm in 100 mM and 500 mM NaCl, respectively.

Steric Repulsion. The adsorbed polymer layer on NPs can lead to an increase in osmotic pressure and elastic repulsion. The repulsive steric interaction energy (E_s) is defined as the sum of osmotic (E_{osm}) and elastic repulsive energies (E_{elas}). The equation of E_{osm} could be expressed as follows:[16, 17]

$$\frac{E_{osm}}{k_B T} = 0,\ 2d \leq h \tag{S7}$$

$$\frac{E_{osm}}{k_B T} = \frac{4\pi R}{v_1}\Phi_{NOM}^2\left(\frac{1}{2} - \chi\right)\left(d - \frac{h}{2}\right)^2, d \leq h \leq 2d \tag{S8}$$

$$\frac{V_{osm}}{k_B T} = \frac{4\pi R}{v_1}\Phi_{NOM}^2\left(\frac{1}{2} - \chi\right)d^2\left(\frac{h}{2d} - \frac{1}{4} - ln\left(\frac{h}{d}\right)\right),\ \text{h<d} \tag{S9}$$

where v_1 is the volume of one solvent molecule (0.03 nm^3), χ is the Flory-Huggins solvency parameter (0.45), d (nm) is the thickness of adsorbed NOM, estimated as half of the difference in the hydrodynamic size of NPs before and after NOM adsorption in 100 mM NaCl (Table S2),[18] ϕ_{NOM} is the effective volume fraction of the adsorbed NOM layer and can be expressed as follows:

$$\Phi_{NOM} = 3\frac{QR^2}{\rho_{NOM}[(d+R)^3 - R^3]} \tag{S10}$$

where Q (mg/m^2) is the maximum adsorbed amount of NOM on NP surface (Table S4). ρ_{NOM} is the density of NOM (assumed to be 1.5 g/cm^3).[19]

The equation of E_{elas} could be expressed as follows:[20]

$$\frac{E_{elas}}{k_B T} = 0, d \leq h \tag{S11}$$

$$\frac{E_{elas}}{k_B T} = \frac{2\pi R}{M_W}\Phi_{NOM}d^2\rho_{NOM}\left[\frac{2}{3} - \frac{1}{6}\left(\frac{h}{d}\right)^3 - \left(\frac{h}{2d}\right) + \left(\frac{h}{d}\right)ln\left(\frac{h}{d}\right)\right],\quad d > h \tag{S12}$$

where M_W (Da) is the molecular weight of NOM.[18] The MWs of bulk NOM and its fractions were estimated as follows: bulk NOM at 12,000 Da, NOM (> 30 kDa) at 50,000 Da, NOM (10−30 kDa) at 20,000 Da, NOM (3−10 kDa) at 6,500 Da, and NOM (< 3 kDa) at 1,500 Da.

Kuhl et al. also developed a steric repulsion model and the equation of E_S could be expressed as follows:[21]

$$E_S = \frac{16\pi KTdR}{35s^3}\left(56\left(\frac{2d}{h}\right)^{\frac{1}{4}} - \frac{40}{11}\left(\frac{h}{2d}\right)^{\frac{11}{4}} + 24\frac{h}{2d} - \frac{840}{11}\right) \quad \text{(S13)}$$

Where s (nm) is the mean distance between anchoring (or grafting) sites on the surface (Table S6).[22] s could be calculated using following equation:

$$s = \sqrt{\frac{Mw}{NA \times Q}} \quad \text{(S14)}$$

where M_W (Da) is the molecular weight of NOM, NA is the Avogadro constant (6.02×10^{23} mol^{-1}), Q (mg/m^2) is the adsorbed amount of NOM on NP surface (Table S3). The corresponding surface coverage (%) of NOM on NPs was shown in Table S4.

Both steric repulsion models could predict the stabilization of pristine NPs by NOM. The calculated interaction energies based on osmotic pressure and elastic repulsion were much higher than those from the secondary steric model by Kuhl et al. Additionally, since the secondary steric repulsion model shared the same parameters as the polymer bridging model, the secondary steric model was used in this study.

Polymer Bridging. Attractive polymer-mediated interactions (i.e. polymer bridging) occur when the polymer's affinity for another surface surpasses a certain threshold. The bridging adhesion energy (E_B) is calculated as follows:[23]

$$E_B = \frac{2\pi R}{s^2 l}\left(l_c h - \frac{h^2}{2} - \frac{l_c^2}{2}\right) \quad \text{(S15)}$$

where l (nm) is a segment length is considered as twice the C-C bond length (0.304 nm), lc is the polymer contour length (Table S2), ε (kT) is the bonding energy per segment (assumed to

be 0.3 kT) and *s* (nm) is the mean distance between anchoring (or grafting) sites on the surface (Table S5).[22]

Combination of DLVO and Steric Model. Conventional DLVO theory considers interactions between "hard" particle surfaces. Some studies have integrated the steric model with the conventional DLVO theory by using the apparent zeta potential for calculations, which is suitable at low ionic strengths.[19, 24] In such conditions, the adsorbed layer thickness is smaller than the Debye length, so adsorbed polymers do not substantially distort the electrical potential distribution in the ionic diffuse layer. However, at high ionic strength, the adsorbed layer thickness may exceed the Debye length, making conventional DLVO theory inapplicable, as it would suggest that electrostatic interactions occur after the contact of adsorbed layers.

Ohshima's electrophoretic theory proposes that the outer surface potential (of the adsorbed layer) is the appropriate electrical potential for predicting electrostatic interactions between "soft" particles coated by polymers.[25, 26] However, Ohshima's theory applies DLVO principles to adsorbed layers and sometimes fails to accurately describe colloidal interactions of polymer-coated particles.[25, 27] Additionally, Ohshima's theory addresses pre-contact DLVO interactions between adsorbed layers, while the steric model describes post-contact particle interactions. Integrating the steric model with Ohshima's DLVO theory is difficult as they start from different zero separation distances.

Therefore, combining the steric model with both conventional DLVO theory and Ohshima's theory at high ionic strengths poses a challenge. Studies have indicated that, at high ionic strength, the effective distance of electrical double layer repulsion between approaching particles may be governed by the adsorbed layer thickness rather than the Debye length.[28] To address this, we used a modified Debye length (effective electrical double layer) in the conventional DLVO theory in this study. The modified Debye length was assumed to be the sum of the NOM adsorption layer thickness and the theoretical k^{-1}.[29, 30]

Table S2. The d and Lc (nm) of bulk NOM and NOM fractions (2 mg/L) on pristine and photoaged NPs, estimated as half of the difference in the hydrodynamic size of NPs before and after NOM adsorption in 100 mM NaCl (Figure 2d). d and Lc (nm) in 500 mM NaCl were assumed to be the same.

d or Lc (nm)	Bulk	> 30 kDa	10-30 kDa	3-10 kDa	< 3 kDa
PS_0	2.81	6.07	4.82	4.29	2.02
PS_2	4.19	6.62	3.87	3.22	1.58
PS_4	4.23	6.92	4.77	4.05	2.95
PS_8	3.63	5.91	4.17	2.92	2.33

Table S3. Adsorption capacity (Q, mg/m²) of bulk NOM and NOM fractions (2 mg/L) on pristine and photoaged NPs, as estimated based on NOM removal conducted in 100 mM NaCl (Figure 2a), and particle size of NPs (Figure 2c). Q in 500 mM NaCl were assumed to be the same.

Q (mg/m^2)	Bulk	> 30 kDa	10-30 kDa	3-10 kDa	< 3 kDa
PS_0	3.35	2.94	2.79	2.72	2.28
PS_2	3.22	2.88	2.86	2.71	2.24
PS_4	2.97	2.78	2.92	2.81	2.1
PS_8	2.51	2.48	3.03	2.84	2.14

Table S4. Surface coverage (%) of bulk NOM and NOM fractions (2 mg/L) on pristine and photoaged NPs, as estimated based on NOM density (assumed to be 1.5 g/cm^3), molecular size, and adsorption amount. NOM molecules were assumed to have a spherical geometry, with their molecular sizes calculated using the online Molecular Weight to Size Calculator (https://nanocomposix.com/pages/molecular-weight-to-size-calculator).

Coverage (%)	Bulk	> 30 kDa	10-30 kDa	3-10 kDa	< 3 kDa
PS_0	114.2	62.1	80.1	113.7	155.2
PS_2	102.9	57.0	77.0	106.0	143.2
PS_4	88.6	51.6	73.6	102.8	125.5
PS_8	72.3	44.4	73.5	100.2	123.3

Table S5. The distance between anchoring sites (s, nm) of bulk NOM and NOM fractions (2 mg/L) on pristine and photoaged NPs, as estimated based on Equation S14. The MWs of bulk NOM and its fractions were estimated as follows: bulk NOM at 12,000 Da, NOM (> 30 kDa) at 50,000 Da, NOM (10−30 kDa) at 20,000 Da, NOM (3−10 kDa) at 6,500 Da, and NOM (< 3 kDa) at 1,500 Da. s in 500 mM NaCl were assumed to be the same.

s (nm)	Bulk	> 30 kDa	10-30 kDa	3-10 kDa	< 3 kDa
PS_0	1.51	5.31	3.45	1.99	1.04
PS_2	2.48	5.37	3.41	2.00	1.05
PS_4	2.59	5.48	3.37	1.96	1.09
PS_8	2.82	5.78	3.31	1.95	1.08

Table S6. Functional groups of pristine and photoaged PS NPs at their respective wavenumbers measured using FTIR.

Wavenumber (cm^{-1})	Functional groups	References
696, 753, and 1028	C–H in-plane bending/aromatic C–H bending	[2, 9, 31, 32]
1452	aromatic ring movement or $–CH_2–$ vibration	[31-34]
1493 and 1601	C=C stretching of the aromatic ring	[31-34]
2850	C–H stretching vibration of the –CH group in the aliphatic segments	[50, 51]
2920	C–H stretching vibration of $–CH_2–$ group in the aliphatic segments	[50, 51]
3026	C–H vibration of the aromatic ring	[53-56]
1717	C=O group	[12]
3453	–OH group	[12]

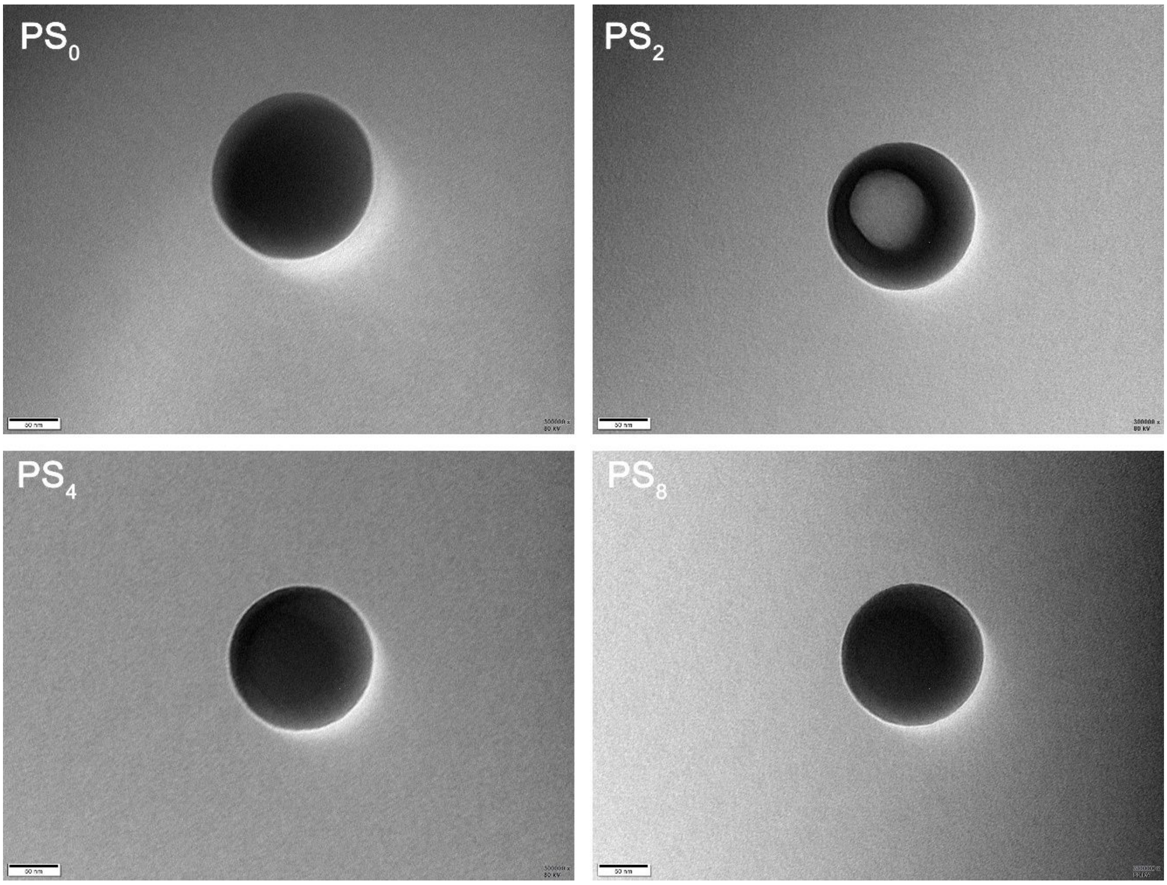

Figure S6. TEM images of pristine and photoaged NPs

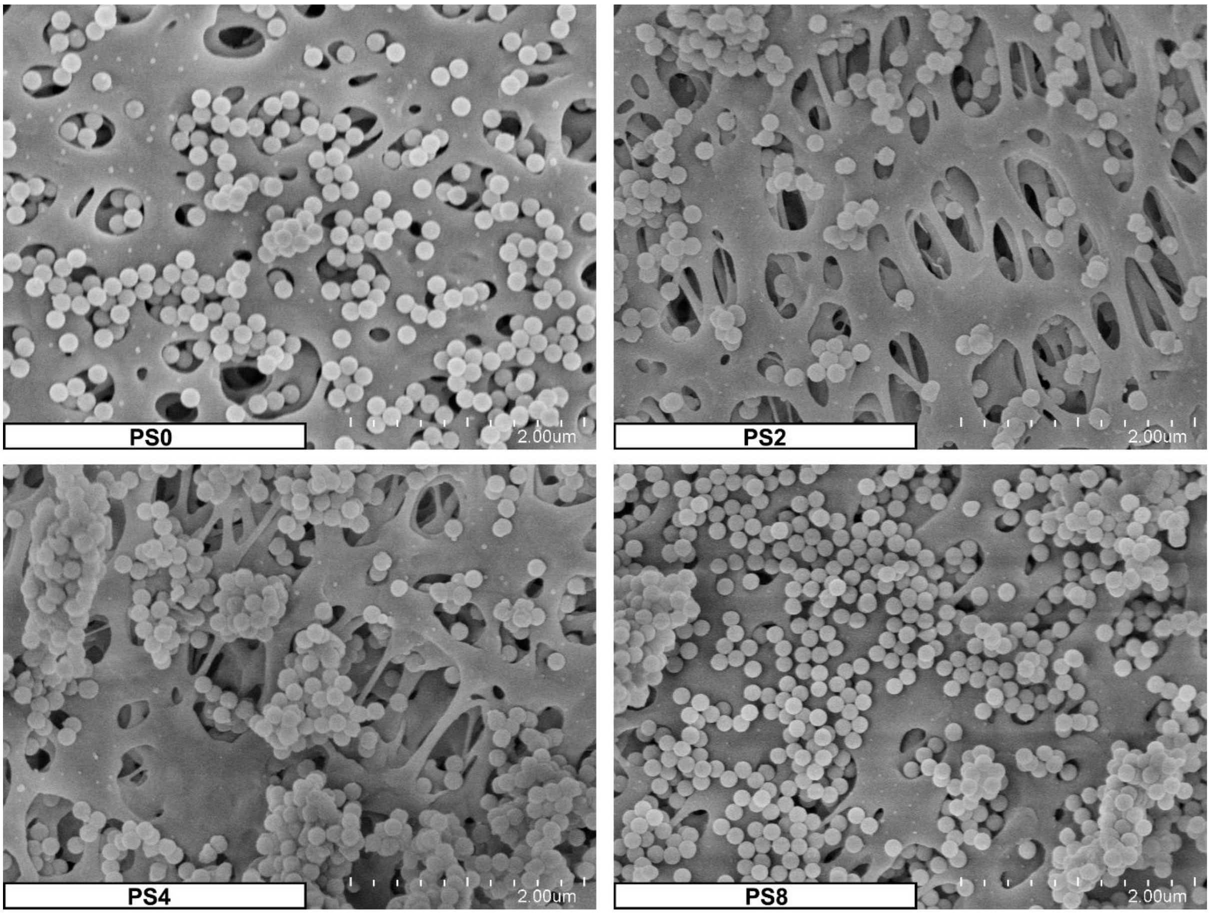

Figure S7. SEM images of pristine and photoaged NPs (NPs were obtained by filtration on membranes and gold sputtering was conducted prior to measurement).

Table S7. Functional groups of bulk NOM and NOM fractions at their respective wavenumbers measured using FTIR.

Wavenumber (cm^{-1})	Functional groups	References
1042 and 1112	C−O stretching of alcohols or carbohydrates	[35, 36]
1388	OH deformation, C−O stretching of phenolic OH, or deprotonated carboxyl groups (COO^-) asymmetric stretching	[35, 37]
1574	aromatic alkenes, conjugated carbonyl, or COO^- asymmetric stretching	[38]
1747	C=O stretch of amide, quinone, or protonated carboxylic acids (COOH)	[39, 40]
3300	–OH stretching	[35, 40, 41]

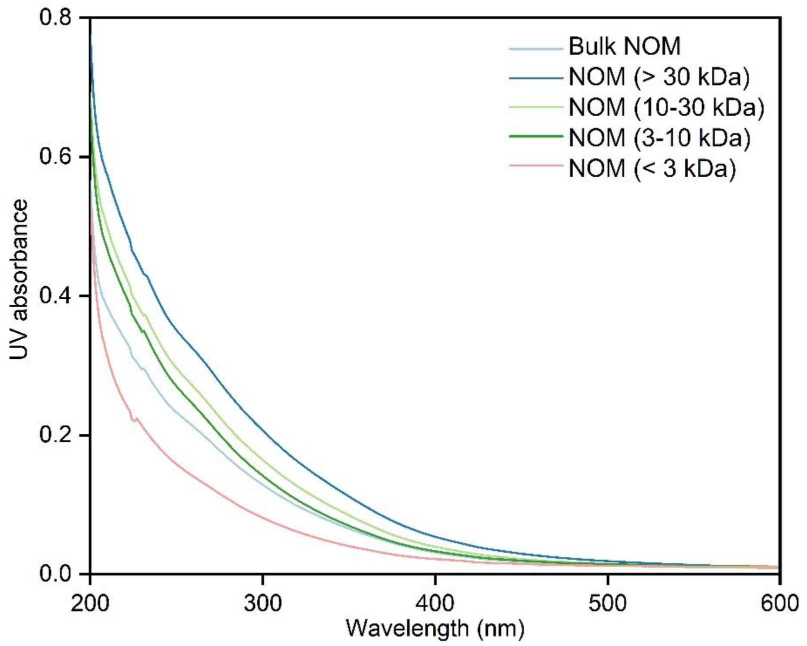

Figure S8. UV absorbance of bulk NOM and different NOM fractions (4 mg C/L).

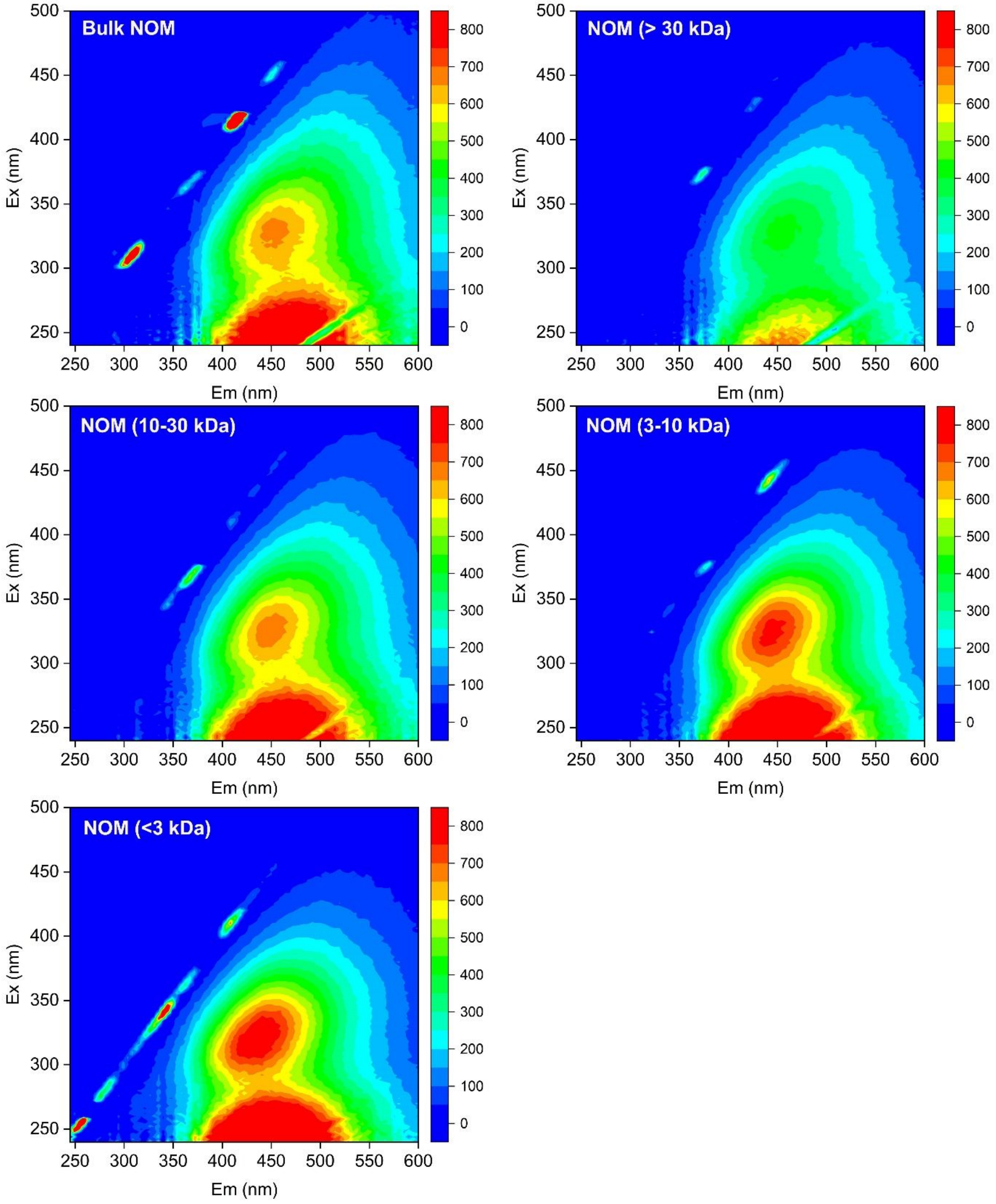

Figure S9. 3D-EEM spectra of bulk NOM and different NOM fractions

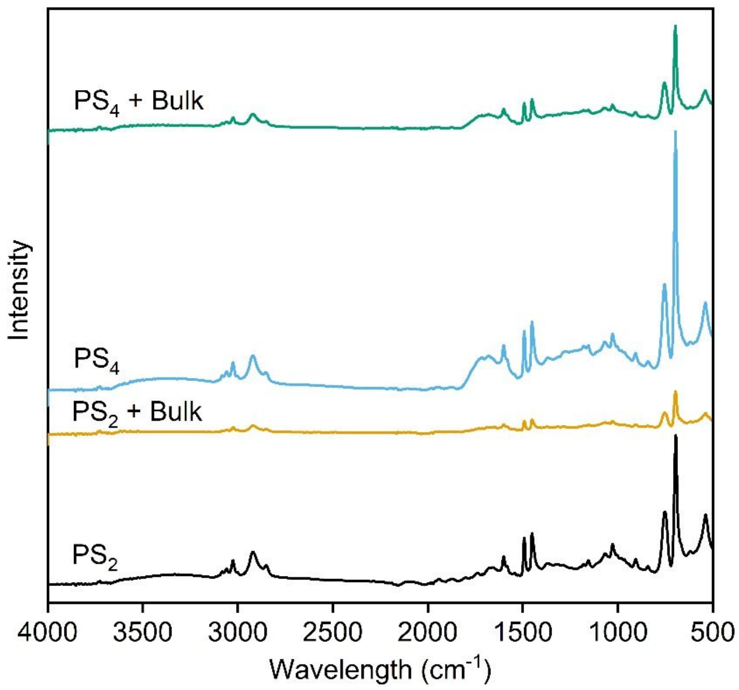

Figure S10. FTIR spectra of PS_2 and PS_4 before and after adsorption with bulk NOM in 100 mM NaCl.

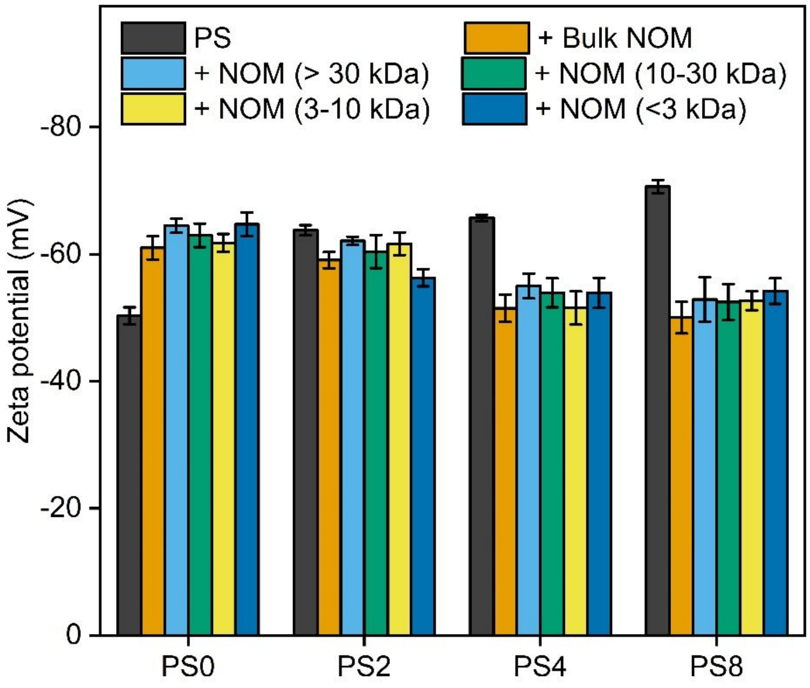

Figure S11. The zeta potential of pristine and photoaged NPs with and without bulk NOM and NOM fractions in 10 mM NaCl. Error bars represent the mean ± 1.96 SE (n = 10).

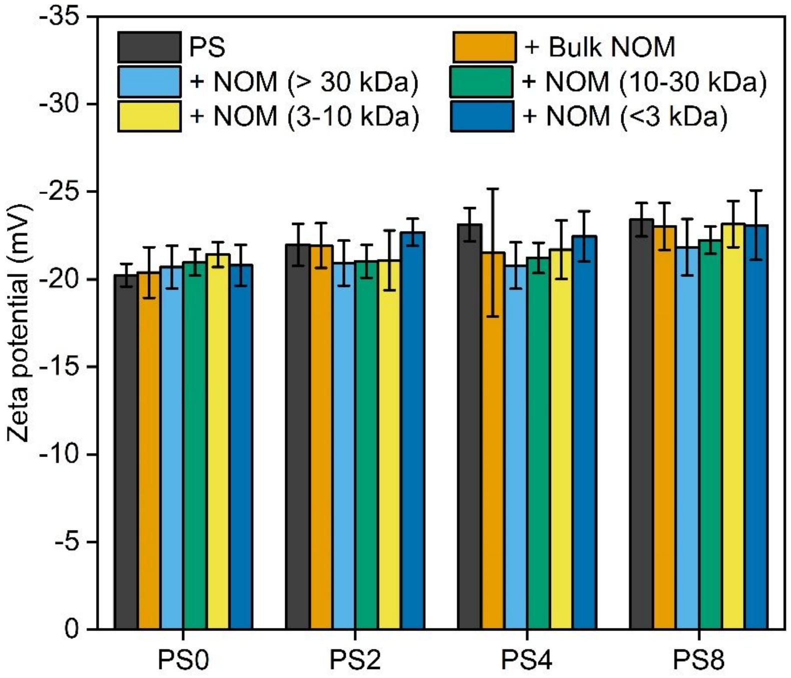

Figure S12. The zeta potential of pristine and photoaged NPs with and without bulk NOM and NOM fractions in 500 mM NaCl. Error bars represent the mean ± 1.96 SE (n = 10).

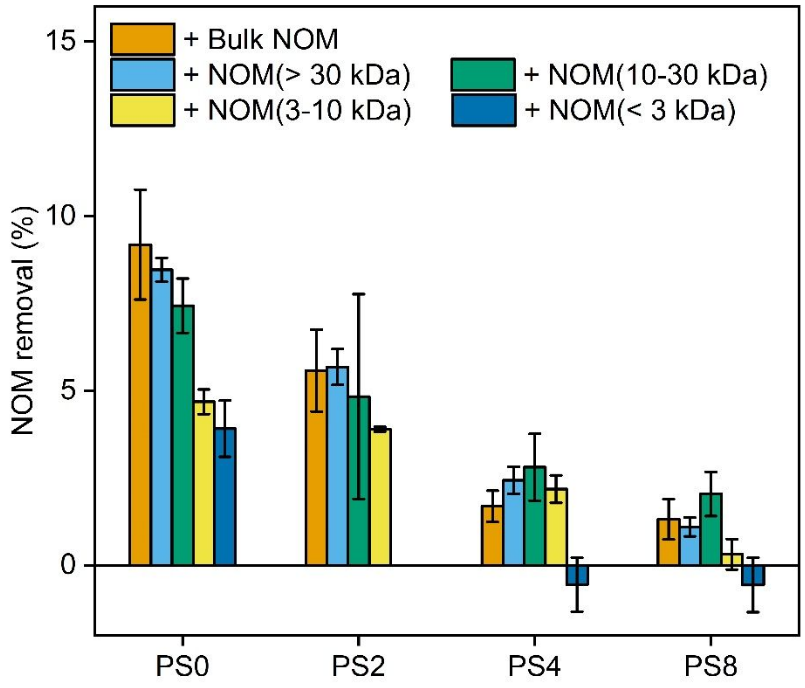

Figure S13. The reduction in UV280 (NOM removal) of bulk NOM and NOM fractions after adsorption on pristine and photoaged NPs in 10 mM NaCl. Error bars represent the mean ± 1.96 SE (n = 2)

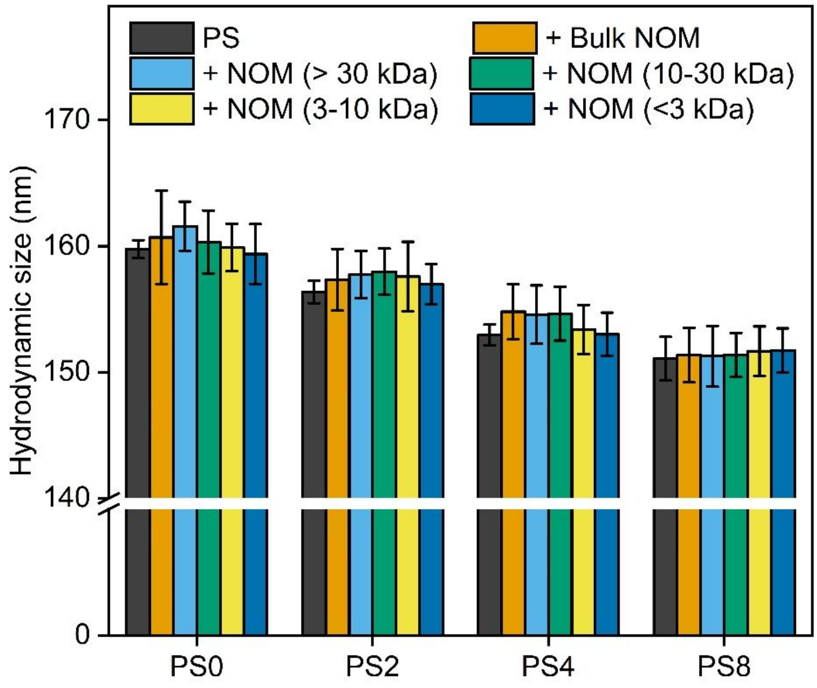

Figure S14. The hydrodynamic size of pristine and photoaged NPs with and without bulk NOM and NOM fractions in 10 mM NaCl. Error bars represent the mean ± 1.96 SE (n = 10)

Result	Name	Status	Repetition	Start time	Temperature	Focus position	Processed runs
					°C	mm	
	N-100 1	Failed	1	08:53:35	25	-0.8	3
	N-100 2	Failed	2	08:55:49	25	-0.8	3
	N-100 3	Failed	3	08:56:34	25	-0.8	3
	N-100 4	Failed	4	08:57:19	25	-0.8	3
	N-100 5	Failed	5	08:58:05	25	-0.8	3
	N-100 6	Failed	6	08:58:50	25	-0.8	3
	N-100 7	Failed	7	08:59:35	25	-0.8	3
	N-100 8	Failed	8	09:00:20	25	-0.8	3
	N-100 9	Failed	9	09:01:05	25	-0.8	3
	N-100 10	Failed	10	09:01:50	25	-0.8	3
	N-100 11	Failed	11	09:02:35	25	-0.8	3
	N-100 12	Failed	12	09:03:20	25	-0.8	3
	N-100 13	Canceled	13	09:04:03	25	-0.8	2
Average							
St.Dev.							
RSD%							

Result	Name	Status	Repetition	Start time	Temperature	Focus position	Processed runs
					°C	mm	
	N-500 1	Failed	1	09:49:33	25	-0.4	3
	N-500 2	Failed	2	09:51:47	25	-0.4	3
	N-500 3	Failed	3	09:52:32	25	-0.4	3
	N-500 4	Failed	4	09:53:17	25	-0.4	3
	N-500 5	Failed	5	09:54:02	25	-0.4	3
	N-500 6	Failed	6	09:54:47	25	-0.4	3
	N-500 7	Failed	7	09:55:32	25	-0.4	3
	N-500 8	Failed	8	09:56:17	25	-0.4	3
	N-500 9	Failed	9	09:57:02	25	-0.4	3
	N-500 10	Failed	10	09:57:48	25	-0.4	3
	N-500 11	Canceled	11	09:58:34	25	-0.4	1
Average							
St.Dev.							
RSD%							

Figure S15. The size characterization results of bulk NOM (10 mg/L) over time at 100 mM and 500 mM NaCl

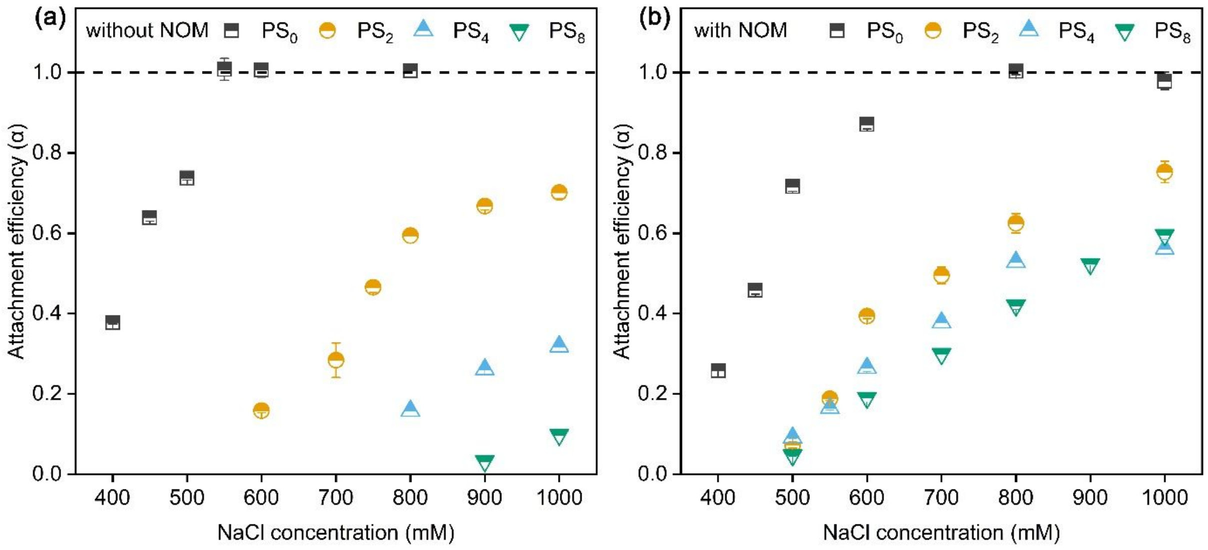

Figure S16. The aggregation kinetics of pristine and photoaged NPs without NOM (a) and with bulk NOM (b). Error bars represent the mean ± SD (n = 2 or 3)

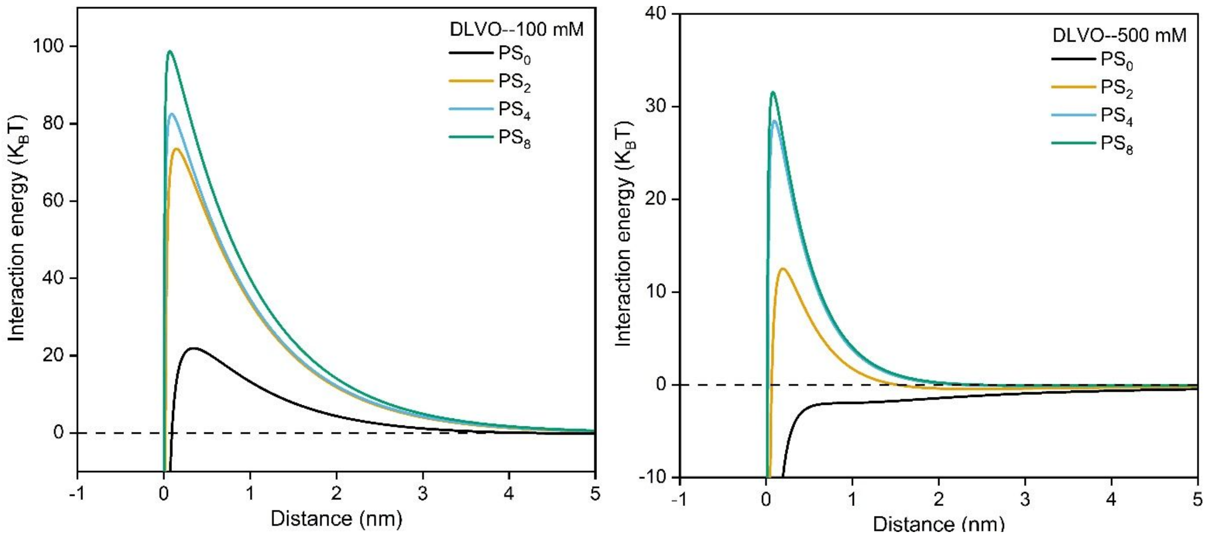

Figure S17. DLVO interaction energy of pristine and photoaged NPs at 100 and 500 mM NaCl solutions.

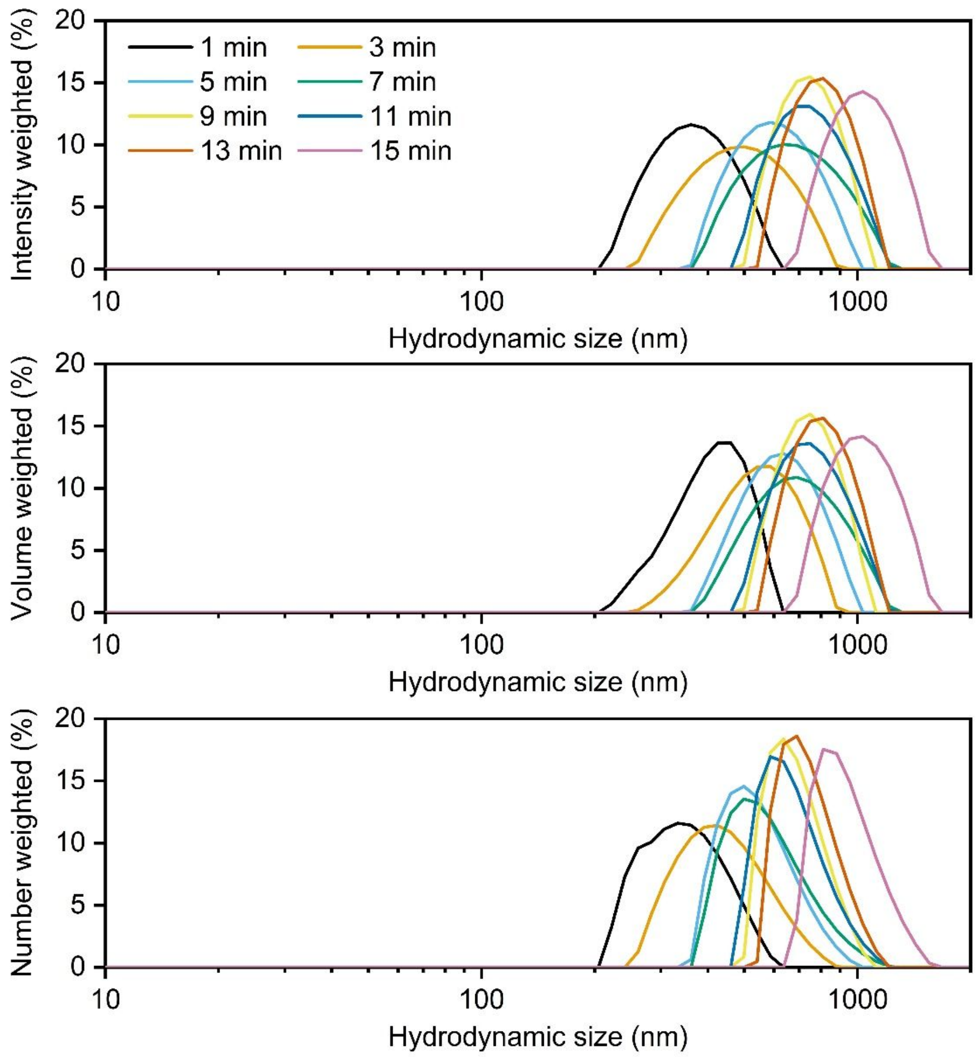

Figure S18. The intensity, volume, and number-weighted size distributions of pristine NPs at 500 mM NaCl over time

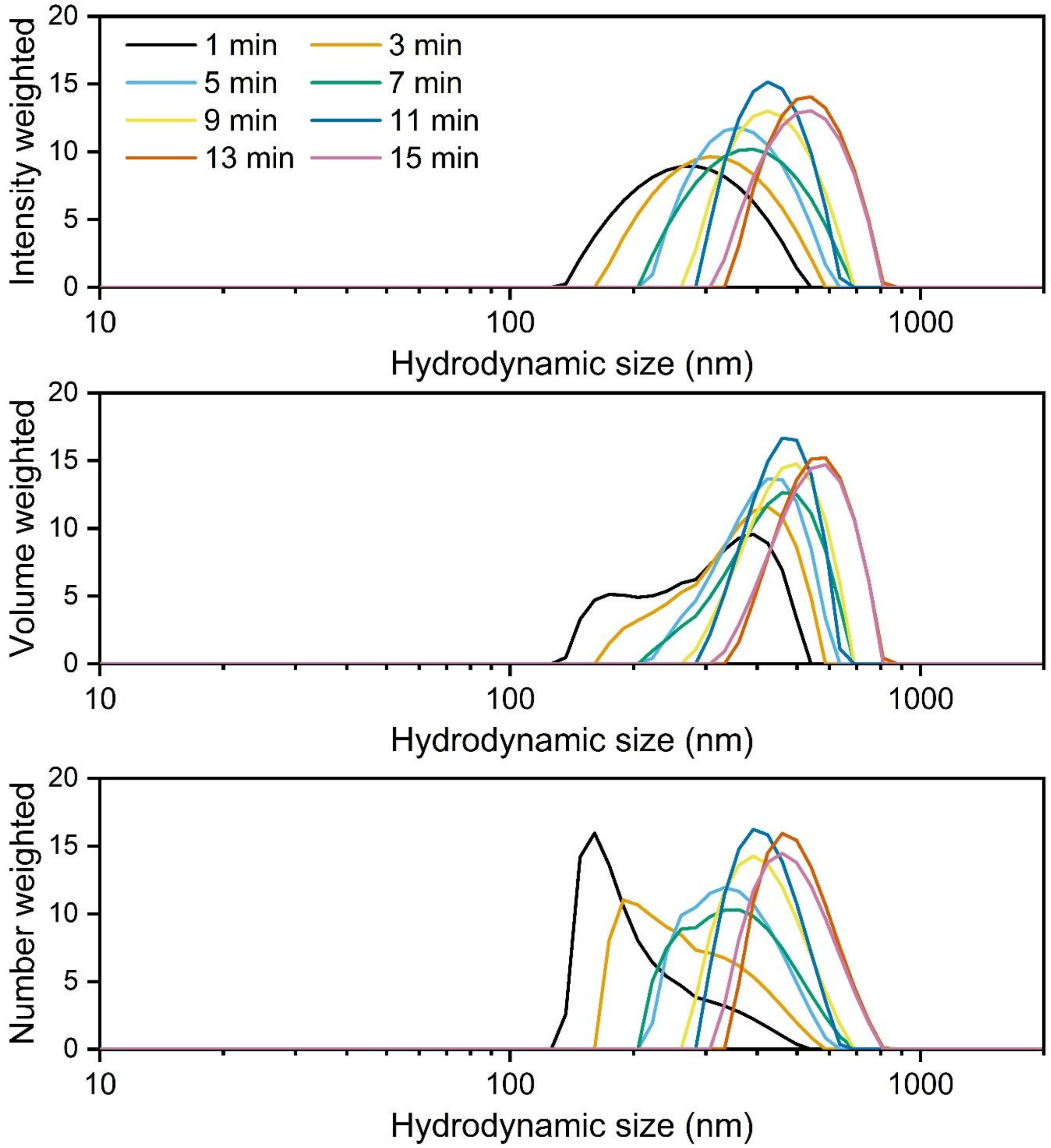

Figure S19. The intensity, volume, and number-weighted size distributions (%) of pristine NPs in the presence of bulk NOM (10 mg/L) at 500 mM NaCl over time.

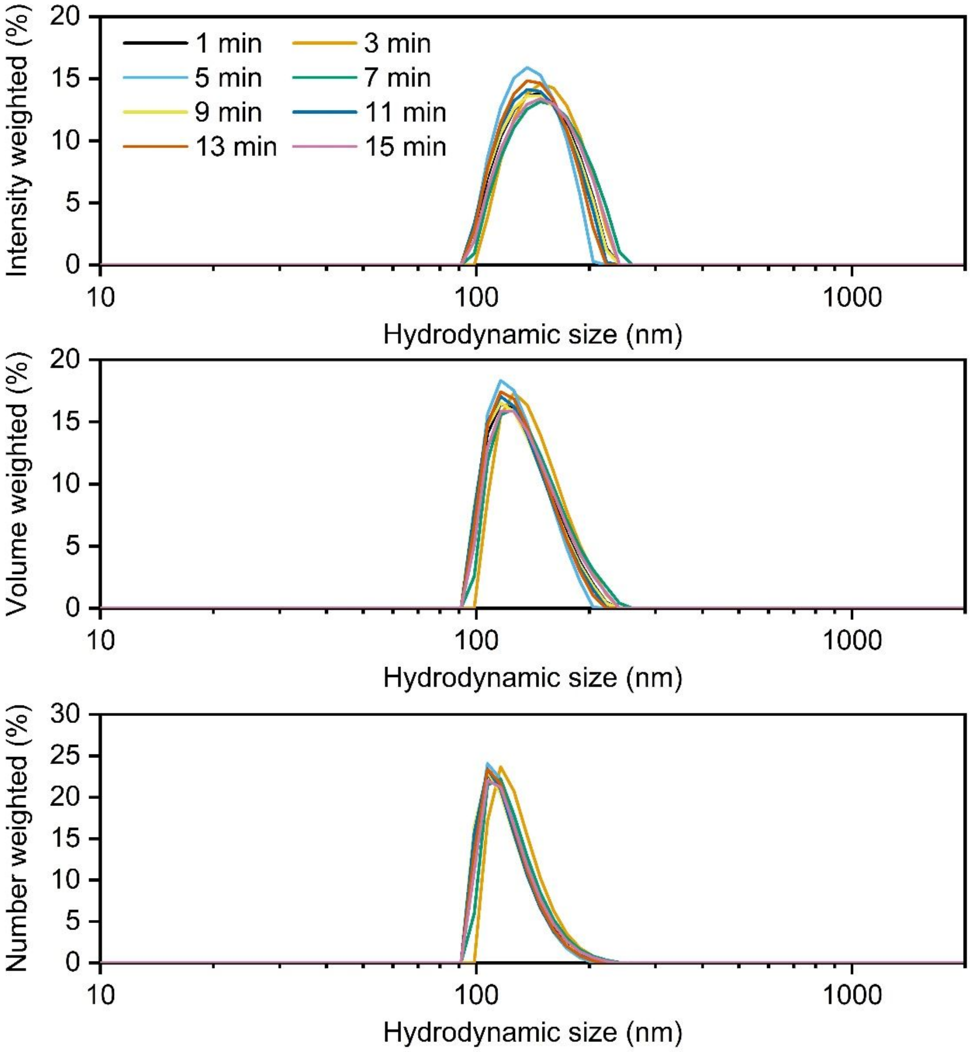

Figure S20. The intensity, volume, and number-weighted size distributions of photoaged NPs (PS_4) at 500 mM NaCl over time

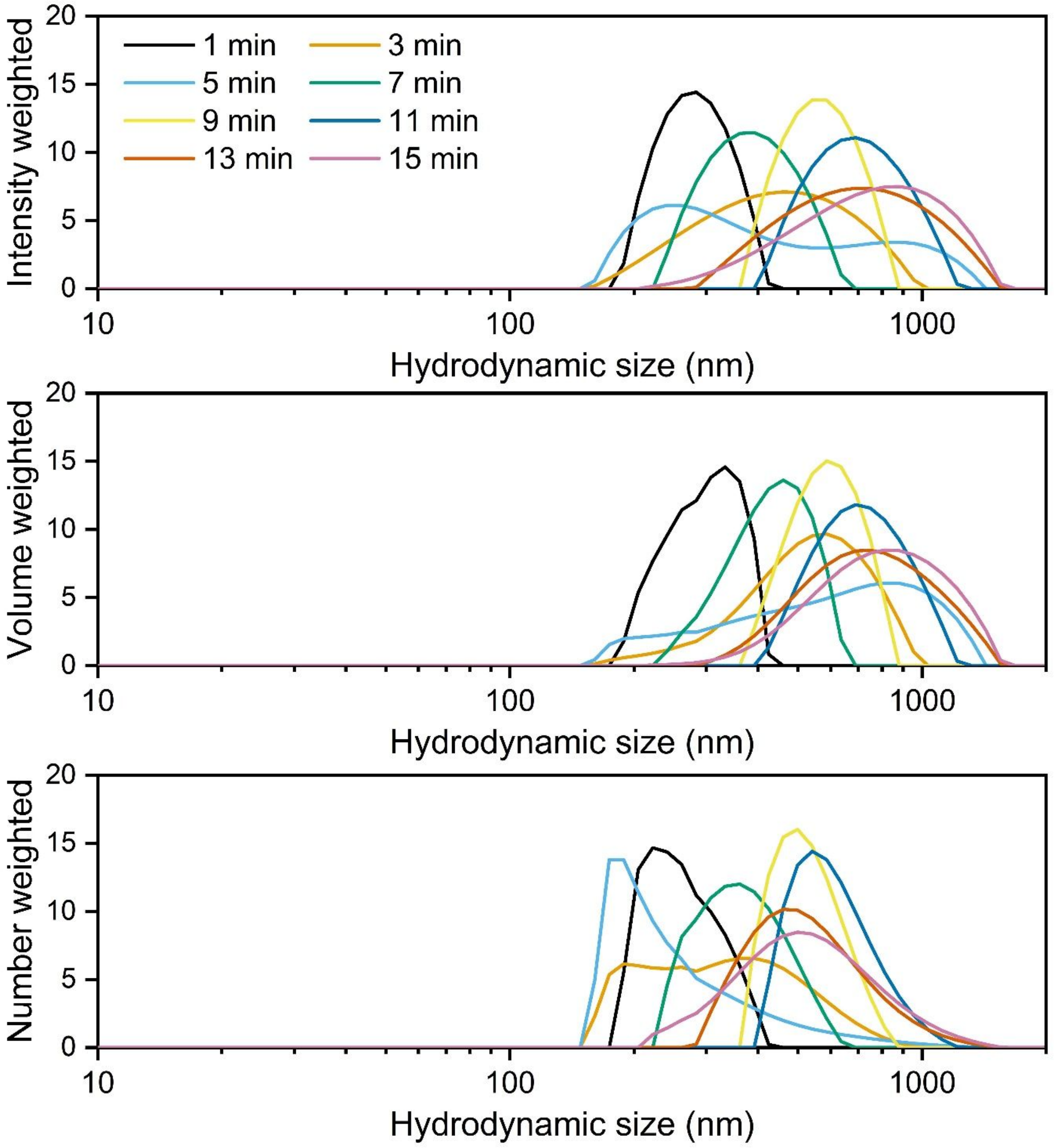

Figure S21. The intensity, volume, and number-weighted size distributions (%) of photoaged NPs (PS_4) in the presence of bulk NOM (10 mg/L) at 500 mM NaCl over time

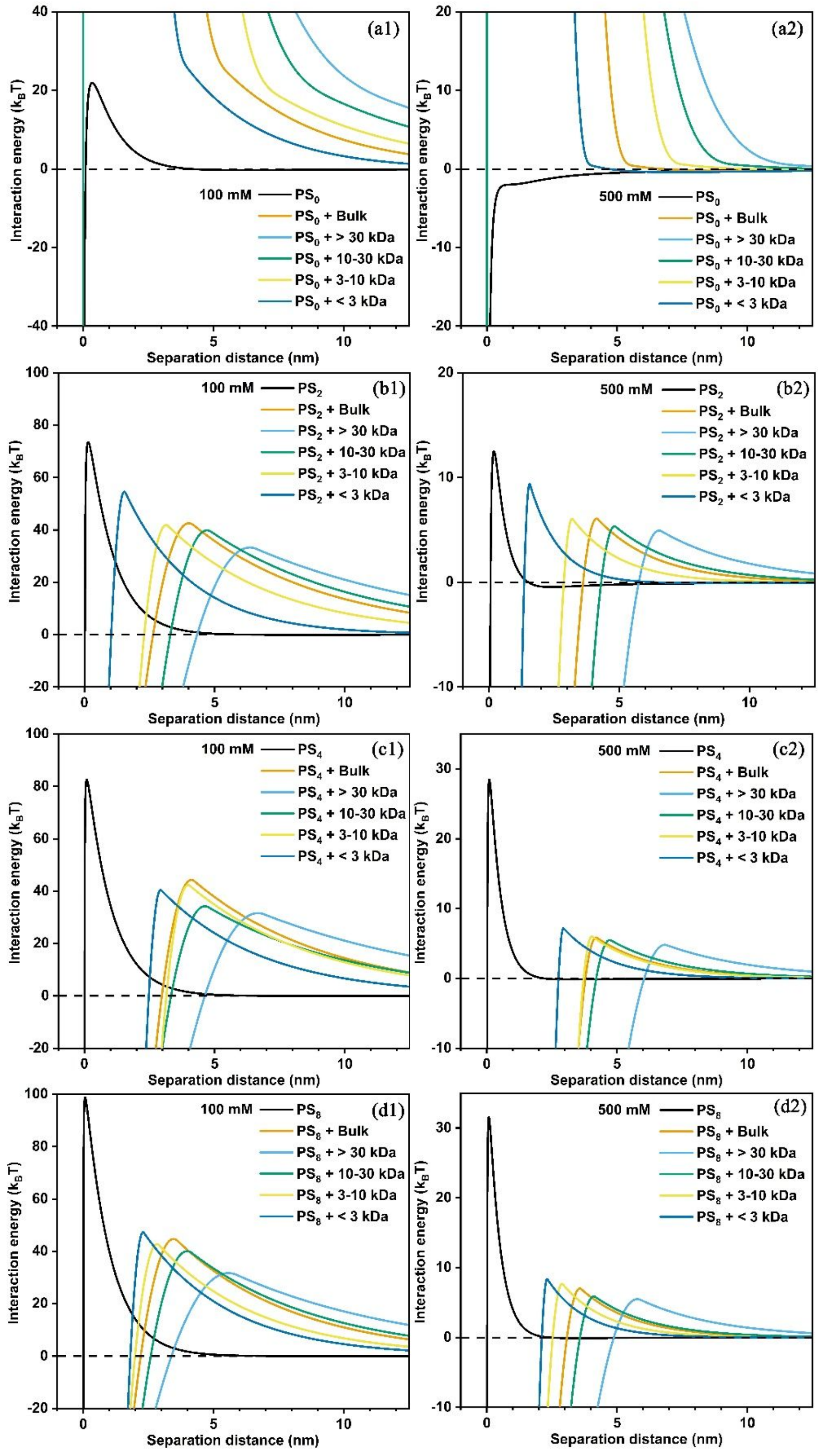

Figure S22. DLVO and steric interaction energy of PS0 (a), PS2 (b), PS4 (c) and PS8 (d) in the absence and presence of bulk NOM and NOM fractions at 100 and 500 mM NaCl solutions

References

1. Xu, Y.; Ou, Q.; Liu, C.; Zhou, X.; He, Q.; Wu, Z.; Huang, R.; Ma, J.; Lu, D.; Huangfu, X., Aggregation and deposition behaviors of dissolved black carbon with coexisting heavy metals in aquatic solution. *Environmental Science: Nano* **2020**, 2773-2784.
2. Liu, Y.; Hu, Y.; Yang, C.; Chen, C.; Huang, W.; Dang, Z., Aggregation kinetics of UV irradiated nanoplastics in aquatic environments. *Water Res* **2019,** *163*, 114870.
3. Liu, Y.; Huang, Z.; Zhou, J.; Tang, J.; Yang, C.; Chen, C.; Huang, W.; Dang, Z., Influence of environmental and biological macromolecules on aggregation kinetics of nanoplastics in aquatic systems. *Water Res* **2020,** *186*, 116316.
4. Wang, J.; Zhao, X.; Wu, A.; Tang, Z.; Niu, L.; Wu, F.; Wang, F.; Zhao, T.; Fu, Z., Aggregation and stability of sulfate-modified polystyrene nanoplastics in synthetic and natural waters. *Environ Pollut* **2021,** *268*, (Pt A), 114240.
5. Yu, S.; Shen, M.; Li, S.; Fu, Y.; Zhang, D.; Liu, H.; Liu, J., Aggregation kinetics of different surface-modified polystyrene nanoparticles in monovalent and divalent electrolytes. *Environ Pollut* **2019,** *255*, (Pt 2), 113302.
6. Shams, M.; Alam, I.; Chowdhury, I., Aggregation and stability of nanoscale plastics in aquatic environment. *Water Res* **2020,** *171*, 115401.
7. Qu, i.; Hwang, Y. S.; Alvarez, P. J. J.; Bouchar, D.; Li, Q., UV Irradiation and humic acid mediate aggregation of aqueous fullerene (nC(60)) nanoparticles. *Environ. Sci. Technol.* **2010,** *44*, (20), 7821–7826.
8. Ter Halle, A.; Jeanneau, L.; Martignac, M.; Jarde, E.; Pedrono, B.; Brach, L.; Gigault, J., Nanoplastic in the North Atlantic Subtropical Gyre. *Environ Sci Technol* **2017,** *51*, (23), 13689-13697.
9. Mao, Y.; Li, H.; Huangfu, X.; Liu, Y.; He, Q., Nanoplastics display strong stability in aqueous environments: Insights from aggregation behaviour and theoretical calculations. *Environ Pollut* **2020,** *258*, 113760.
10. Chen, C.; Huang, W., Aggregation kinetics of nanosized activated carbons in aquatic environments. *Chemical Engineering Journal* **2017,** *313*, 882-889.
11. Xu, Y.; Ou, Q.; He, Q.; Wu, Z.; Ma, J.; Huangfu, X., Influence of dissolved black carbon on the aggregation and deposition of polystyrene nanoplastics: Comparison with dissolved humic acid. *Water Res* **2021,** *196*, 117054.
12. Ou, Q.; Xu, Y.; He, Q.; Wu, Z.; Ma, J.; Huangfu, X., Deposition behavior of dissolved black carbon on representative surfaces: Role of molecular conformation. *Journal of Environmental Chemical Engineering* **2021,** *9*, (5), 105921.
13. Yeap, S. P.; Lim, J.; Ngang, H. P.; Ooi, B. S.; Ahmad, A. L., Role of Particle-Particle Interaction Towards Effective Interpretation of Z-Average and Particle Size Distributions from Dynamic Light Scattering (DLS) Analysis. *J Nanosci Nanotechnol* **2018,** *18*, (10), 6957-6964.
14. Huangfu, X.; Xu, Y.; Liu, C.; He, Q.; Ma, J.; Ma, C.; Huang, R., A review on the interactions between engineered nanoparticles with extracellular and intracellular polymeric substances from wastewater treatment aggregates. *Chemosphere* **2019,** *219*, 766-783.
15. Baalousha, M.; Nur, Y.; Romer, I.; Tejamaya, M.; Lead, J. R., Effect of monovalent and divalent cations, anions and fulvic acid on aggregation of citrate-coated silver nanoparticles. *Sci Total Environ* **2013,** *454-455*, 119-31.
16. Fritz, G.; Schädler, V.; Willenbacher, N.; Wagner, N. J., Electrosteric Stabilization of Colloidal Dispersions. *Langmuir* **2002,** *18*, (16), 6381-6390.
17. Phenrat, T.; Saleh, N.; Sirk, K.; Kim, H.-J.; Tilton, R. D.; Lowry, G. V., Stabilization of aqueous nanoscale zerovalent iron dispersions by anionic polyelectrolytes: adsorbed anionic

polyelectrolyte layer properties and their effect on aggregation and sedimentation. *J. Nanopart. Res.* **2008,** *10*, (5), 795-814.
18. Wang, D.; Jin, Y.; Jaisi, D. P., Effect of Size-Selective Retention on the Cotransport of Hydroxyapatite and Goethite Nanoparticles in Saturated Porous Media. *Environ. Sci. Technol.* **2015,** *49*, (14), 8461-8470.
19. Liang, W.; Zhang, W.; Shao, X.; Gong, K.; Su, C.; Zhang, W.; Peng, C., Organic matters adsorbed on goethite inhibited the heterogeneous aggregation and adsorption of CdSe quantum dots: Experiments and extended DLVO theory. *J Hazard Mater* **2024,** *467*, 133769.
20. Song, J. E.; Phenrat, T.; Marinakos, S.; Xiao, Y.; Liu, J.; Wiesner, M. R.; Tilton, R. D.; Lowry, G. V., Hydrophobic Interactions Increase Attachment of Gum Arabic- and PVP-Coated Ag Nanoparticles to Hydrophobic Surfaces. *Environ. Sci. Technol.* **2011,** *45*, (14), 5988-5995.
21. Kuhl, T.; Leckband, D.; Lasic, D.; Israelachvili, J., Modulation of interaction forces between bilayers exposing short-chained ethylene oxide headgroups. *Biophysical Journal* **1994,** *66*, (5), 1479-1488.
22. Ji, Y.; Lu, Q.; Liu, Q.; Zeng, H., Effect of solution salinity on settling of mineral tailings by polymer flocculants. *Colloids and Surfaces A: Physicochemical and Engineering Aspects* **2013,** *430*, 29-38.
23. Israelachvili, J. N., *Intermolecular and surface forces*. Academic press: 2011.
24. Wang, D.; Jin, Y.; Jaisi, D. P., Effect of Size-Selective Retention on the Cotransport of Hydroxyapatite and Goethite Nanoparticles in Saturated Porous Media. *Environ Sci Technol* **2015,** *49*, (14), 8461-70.
25. Lin, S.; Wiesner, M. R., Theoretical investigation on the interaction between a soft particle and a rigid surface. *Chemical Engineering Journal* **2012,** *191*, 297-305.
26. Ohshima, H., Electrophoretic mobility of soft particles. *Colloids Surfaces A: Physicochemical Engineering Aspects* **1995,** *103*, (3), 249-255.
27. Kerchove, A. J. d.; Elimelech, M., Relevance of Electrokinetic Theory for “Soft” Particles to Bacterial Cells: Implications for Bacterial Adhesion. *Langmuir* **2005,** *21*, 6462-6472.
28. Tiller, C. L.; O’Melia, C. R., Natural organic matter and colloidal stability: models and measurements. *Colloids and Surfaces A: Physicochemical and Engineering Aspects* **1993,** *73*, 89-102.
29. Okubo, T., Effect of Neutral Polymers on the Ordering of Monodispersed Polystyrene Spheres. *Journal of the Chemical Society, Faraday Transactions 1: Physical Chemistry in Condensed Phases* **1987,** *83*, (8), 2497-2504.
30. Piccinini, E.; Alberti, S.; Longo, G. S.; Berninger, T.; Breu, J.; Dostalek, J.; Azzaroni, O.; Knoll, W., Pushing the Boundaries of Interfacial Sensitivity in Graphene FET Sensors: Polyelectrolyte Multilayers Strongly Increase the Debye Screening Length. *The Journal of Physical Chemistry C* **2018,** *122*, (18), 10181-10188.
31. Song, F.; Li, T.; Hur, J.; Shi, Q.; Wu, F.; He, W.; Shi, D.; He, C.; Zhou, L.; Ruan, M.; Cao, Y., Molecular-level insights into the heterogeneous variations and dynamic formation mechanism of leached dissolved organic matter during the photoaging of polystyrene microplastics. *Water Res* **2023,** *242*, 120114.
32. Chercoles Asensio, R.; San Andres Moya, M.; de la Roja, J. M.; Gomez, M., Analytical characterization of polymers used in conservation and restoration by ATR-FTIR spectroscopy. *Anal Bioanal Chem* **2009,** *395*, (7), 2081-96.
33. Ho, W. K.; Law, J. C.; Zhang, T.; Leung, K. S., Effects of Weathering on the Sorption Behavior and Toxicity of Polystyrene Microplastics in Multi-solute Systems. *Water Res* **2020,** *187*, 116419.

34. Liu, Y.; Yue, T.; Liu, L.; Zhang, B.; Feng, H.; Li, S.; Liu, X.; Dai, Y.; Zhao, J., Molecular assembly of extracellular polymeric substances regulating aggregation of differently charged nanoplastics and subsequent interactions with bacterial membrane. *J Hazard Mater* **2023,** *457*, 131825.
35. Chen, W.; Qian, C.; Liu, X. Y.; Yu, H. Q., Two-dimensional correlation spectroscopic analysis on the interaction between humic acids and TiO2 nanoparticles. *Environmental Science and Technology* **2014,** *48*, (19), 11119-11126.
36. Li, Y.; Gong, X.; Sun, Y.; Shu, Y.; Niu, D.; Ye, H., High molecular weight fractions of dissolved organic matter (DOM) determined the adsorption and electron transfer capacity of DOM on iron minerals. *Chemical Geology* **2022,** *604*.
37. Mudunkotuwa, I. A.; Grassian, V. H., Biological and environmental media control oxide nanoparticle surface composition: the roles of biological components (proteins and amino acids), inorganic oxyanions and humic acid. *Environmental Science: Nano* **2015,** *2*, (5), 429-439.
38. Han, L.; Yang, Y.; Sun, K.; Zhang, B.; Chen, Y.; Fang, L.; Xing, B., Different mechanisms driving the preferential adsorption of dissolved organic matter by goethite and montmorillonite. *Chemical Geology* **2021,** *585*, 120560.
39. Chang, R.; Mylotte, R.; Hayes, M.; Mclnerney, R.; Tzou, Y., A comparison of the compositional differences between humic fractions isolated by the IHSS and exhaustive extraction procedures. *Naturwissenschaften* **2014,** *101*, 197-209.
40. Li, Z.; Shakiba, S.; Deng, N.; Chen, J.; Louie, S. M.; Hu, Y., Natural Organic Matter (NOM) Imparts Molecular-Weight-Dependent Steric Stabilization or Electrostatic Destabilization to Ferrihydrite Nanoparticles. *Environ Sci Technol* **2020,** *54*, (11), 6761-6770.
41. Coates, J., Interpretation of infrared spectra, a practical approach. *Encyclopedia of analytical chemistry* **2000,** *12*, 10815-10837.
